# Multicolor Upconversion Förster Resonant Energy Transfer Using Optimized Yb@YbTm Core@Shell Nanoparticles

**DOI:** 10.1021/acsnano.5c13869

**Published:** 2025-11-24

**Authors:** Grzegorz Bękarski, Katarzyna Prorok, František Štětina, Małgorzata Misiak, Hans H. Gorris, Artur Bednarkiewicz

**Affiliations:** † Institute of Low Temperature and Structure Research, 215275 Polish Academy of Sciences, ul. Okólna 2, 50-422 Wroclaw, Poland; ‡ Department of Biochemistry, Faculty of Science, 161954 Masaryk University, Kamenice 5, 625 00 Brno, Czech Republic

**Keywords:** upconversion, FRET, nanomaterials, multiplexing, sensing

## Abstract

Upconverting nanoparticles (UCNPs) have emerged as promising alternative donors for resonance energy transfer (FRET)-based biosensing. However, employing UCNPs in FRET assays remains challenging because they display relatively small absorption cross sections and are relatively large as compared to the Förster distance. Thousands of individual donor ions in each UCNP are located within various distances from surface-bound acceptors, complicating the data analysis. While previous studies have explored how the composition and architecture of UCNPs influence FRET, many reports remain qualitative, and multicolor UC-FRET systems involving a single donor and multiple acceptors are less commonly studied than single-donor-single-acceptor systems. To address these challenges, we synthesized UCNPs with an absorbing core (Yb^3+^-doped)/active shell (Yb^3+^, Tm^3+^-doped) nanoparticles systematically varying Tm^3+^ concentrations to optimize the FRET efficiency to surface-bound organic acceptors. A shell composition containing 4% Tm^3+^ yielded the highest FRET efficiency. Moreover, four distinct ATTO dyes showing spectral overlap with the blue emission of Tm^3+^ were used as acceptor dyes on the surface of UCNPs to evaluate FRET efficiencies in spectral and time domains. The differentiation of the four ATTO dyes on one type of upconverting donor nanoparticles using a simple ratiometric approach lays the foundation for the design of multiplexed bioassays. Our results offer a strategy for improving UC-FRET sensitivity through smart core–shell UCNPs designs, donor concentration tuning, and provide important insights into the rational design of more efficient, multicolor, and wash-free UC biosensing platforms.

## Graphical abstract

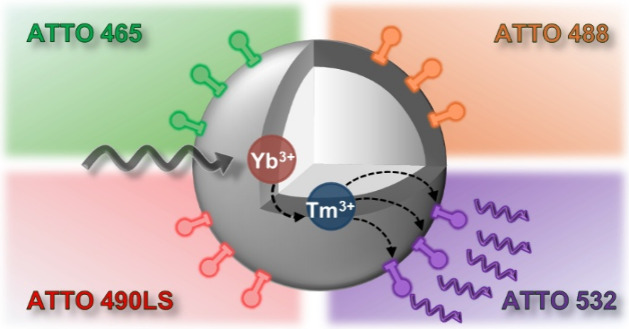

Conventional molecular Förster resonance energy transfer (FRET) is a nonradiative energy transfer process that relies on dipole–dipole interactions between an energy donor (D) and an energy acceptor (A) molecule.^1,2^ FRET has found numerous applications, particularly in biorelated fields such as immunoassays, pH sensing, monitoring of configurational changes of proteins, tracking dynamic processes within live cells in real time, ion detection, and multiplexed analyses.^3–16^ FRET-based biosensors provide high sensitivity; however, they also face significant limitations related to photophysical properties of organic donors and acceptors. Commonly used organic dyes suffer from photobleaching and spectral cross-talk between numerous exogenous and endogenous fluorescent molecules due to their broad absorption and emission bands.^17^ This ultimately leads to elevated unspecific background fluorescence, enforcing complicated detection, correction, and analysis of distinct FRET signals.^18^ Moreover, organic dyes are highly sensitive to their local chemical environment; therefore, the variations in temperature, pH, viscosity, or ion strength of solution can significantly affect the accuracy and reliability of FRET measurements.^11,19–21^

Compared to organic donor dyes, upconverting nanoparticles offer several advantages suitable for FRET bioapplications. Their efficient anti-Stokes emission, photostability, relative insensitivity to chemical environment, narrowband absorption and emission spectra, and long-luminescence lifetimes minimize background signals and spectral cross-talk, thereby improving signal-to-noise (S/N) ratio and enhancing the sensitivity of bioassays.^22–25^ The UCNPs are typically excited by the photoexcitation of Yb^3+^ sensitizers at 980 nm, where no autofluorescence background is generated. The absorbed energy is stored in the long-lived ^2^F_5/2_ energy level of the Yb^3+^ network, leading to efficient energy migration throughout the nanoparticle volume. The Yb^3+^ network is responsible for efficient energy absorption and energy transfer upconversion (ETU) to activator ions such as Er^3+^, Tm^3+^, or Ho^3+^. Despite these promising features, application of UCNP in FRET still remains challenging. First of all, the excited Yb^3+^ ions continuously repopulate activator ions as soon as they nonradiatively transferred their energy to acceptors, making them less responsive to acceptor presence in time domain.^26–29^ Moreover, in contrast to molecular dyes (size of ∼1–2 nm), UCNPs donor nanoparticles typically have diameters of tens of nanometers (commonly ∼30 nm) and contain hundreds to thousands of individual donor ions. Those donor ions are surrounded by a ca. 10-fold larger amount of sensitizer ions (e.g., Yb^3+^ ions), which more effectively absorb, store, and transfer energy throughout UCNPs volume to populate excited states of the donor ions. In consequence, an UCNP cannot be described as a single donor unit as a whole, but rather each Tm^3+^-dopant ion constitutes an individual potential FRET donor site or an individual emitting center. Unlike molecular FRET, where a single donor interacts in principle with a single acceptor, in upconversion-FRET (UC-FRET), many acceptor molecules can be attached to the relatively large nanoparticle surface leading to energy transfer from only those donor ions that are located close to the surface. Due to the size of UCNP and relatively short Förster distance found for lanthanide donors (*R*_0_ typically does not exceed 5 nm), only a fraction of these dopant ions - the ones located near the nanoparticle surface, can effectively participate in FRET.^30–32^ Simple estimations indicate more than 60% of the emitting lanthanide ions are located too deep within the volume of typical UCNP (ca. 4 nm below the surface) to fall into Förster range.^33^

The concept of using core@shell UCNPs, where the donor ions are located only in the shell and thus close to the surface acceptor, to improve FRET efficiency was first suggested by Bednarkiewicz et al.^33^ Similar experimental observations were also reported by Bhuckory et al.^28^ and Siefe et al.^34^ To further circumvent these issues, Pilch et al.^32^ designed an UCNP architecture with a core consisting of NaYF_4_:Yb^3+^ and a shell consisting of NaYF_4_:Yb^3+^,Er^3+^ that is more efficient for FRET than the standard NaYF_4_:Yb^3+^,Er^3+^ homogenous structure. Recent studies combining experimental designing and theoretical modeling confirmed that an additional inert shell can suppress surface quenching and preserve donor emission, leading to optimal performance in core@shell@shell structures.^31^ While Tm^3+^- based UCNPs have emerged as particularly interesting donor systems due to their distinct emission bands and NIR emission,^35–37^ the impact of Tm^3+^ concentration on FRET efficiency has not yet been investigated systematically.

Although there is a common agreement that the most efficient donor UCNPs must incorporate donor ions near the nanocrystal's surface, the complex mechanism of concentration and pump power dependent ETU, surface and concentration quenching, and distribution of dopants are still leaving many unanswered questions. Previous studies mostly focused on Yb^3+^/Er^3+^ doped mainly due to their high brightness and relatively high quantum yield.^28,31,32,34^ However, the brightness alone is not the most important figure of merit when FRET efficiency is a central feature of interest. In contrast to Er^3+^-doped UCNPs, Tm^3+^-doped UCNPs display a wider spectral gap between the emission bands present at approximately 480 nm (^1^G_4_ → ^3^H_6_) and 650 nm (^1^G_4_ → ^3^F_4_). Therefore, using the 480 nm band as the donor emission, at least a few different acceptor dyes can emit without spectral overlap with Tm^3+^ emission bands, potentially enabling multiplexed FRET detection and sensing.

In this study, we investigated the concentration-dependent quenching effects of fluorescent dyes on Tm^3+^ ions located in the shell of the UCNPs in order to understand and quantify FRET. Specifically, we studied the impact of an increased concentration of Tm^3+^ dopant in UCNPs of type NaYF_4_:Yb^3+^@NaYF_4_:Yb^3+^, *x*%Tm^3+^ on multiplexed FRET performance for four distinct ATTO dyes (i.e., ATTO 465, ATTO 488, ATTO 490LS, and ATTO 532). The multiplexing application of UC-FRET was further investigated using various methods—emission spectra, bandpass filter-based microtiter plate reading, and kinetics of upconversion were critically evaluated aiming to find an optimum balance between assay sensitivity, technical feasibility, and multiplexing capabilities.

## Results and Discussion

### Characterization of Nanoparticles

In order to investigate the influence of Tm^3+^ ions concentration on UC-FRET efficiency, we first synthesized three batches of NaYF_4_:50%Yb^3+^ core nanoparticles using a thermal decomposition method. All of them exhibited a hexagonal crystallographic phase (Supporting Information, Figure S1). Their morphology and size were studied using transmission electron microscopy (TEM; Supporting Information, Figure S2). All core nanoparticles were spherical, with diameters of 34.4 ± 1.8, 32.3 ± 1.8, and 32.6 ± 1.4 nm. The slight differences in core sizes can be attributed to minor variations during the synthesis process. Each batch of core nanoparticles was then coated with a NaYF_4_:20%Yb^3+^, *x*% Tm^3+^ shell, where *x* was equal to 0.5, 1, 2, 4, and 6%. The hexagonal structure of the resulting core@shell nanoparticles was confirmed using X-ray diffraction (XRD; Supporting Information, Figure S3) and their sizes were determined via TEM to be 37.3 ± 1.8, 37.5 ± 1.2, 37.6 ± 1.5, 37.0 ± 1.4 and 40.5 ± 1.6 nm, respectively (Figure 1c and Supporting Information, Figure S4). Based on these measurements, the average shell thicknesses were estimated to be 1.5, 2.6, 2.6, 2.2 and 3.1 nm, respectively.

**Figure 1:**
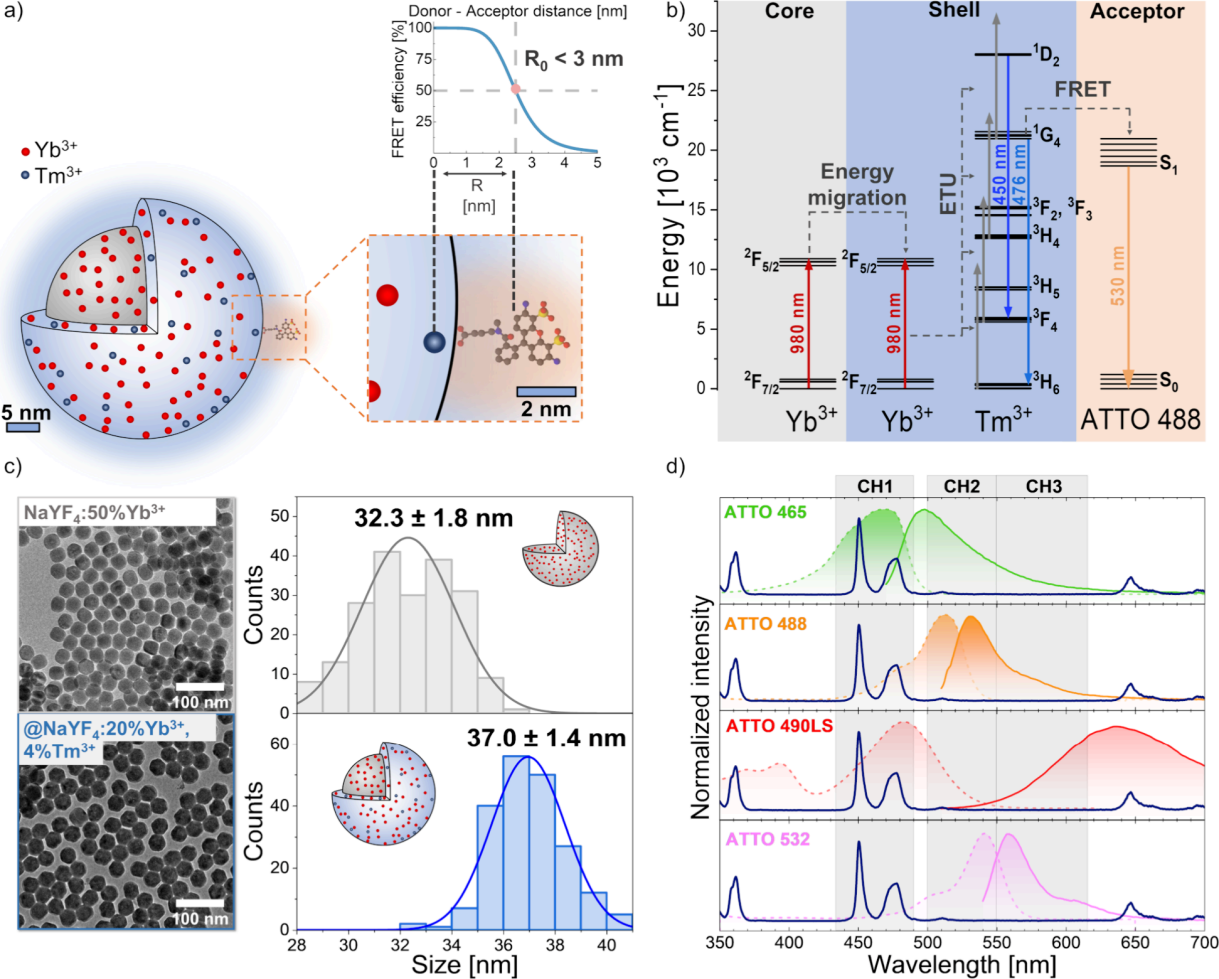
Experimental design and characterization of UCNP donor–acceptor combinations. (a) Schematic representation of upconversion-FRET with Tm^3+^-doped UCNPs conjugated with ATTO 488 dye molecules, (b) energy diagram of UC-FRET, (c) representative TEM images of core NaYF_4_:50%Yb^3+^ (gray) and core@shell NaYF_4_:50%Yb^3+^@NaYF_4_:20%Yb^3+^, 4%Tm^3+^ (blue) with histograms of corresponding diameters, and (d) spectral overlap between the emission of Tm^3+^-doped nanoparticles and the absorption (dashed lines) and emission (full lines) of four acceptor dyes; additionally, three spectral detection channels (CH1, CH2, and CH3) determined by respective bandpass filters are shown.

### Optimization of Tm^3+^ Dopant Concentration in the Shell

As-synthesized UCNPs were redispersed in chloroform and evaluated under 980 nm laser excitation using steady-state emission (Figure 2a) and luminescence lifetime measurements (Supporting Information, Figure S5). Characteristic Tm^3+^ emission bands can be observed ^1^I_6_ → ^3^F_4_ at 344 nm, ^1^D_2_ → ^3^H_6_ at 360 nm, a hypersensitive band ^1^D_2_ → ^3^F_4_ at 450 nm, ^1^G_4_ → ^3^H_6_ at 476 nm, ^1^G_4_ → ^3^F_4_ at 646 nm, and some minor bands as shown in the emission spectra. The architecture of NaYF_4_:50%Yb^3+^@NaYF_4_:20%Yb^3+^,2%Tm^3+^ UCNPs was recognized as the brightest because it showed the most efficient luminescence intensity of UV and blue light (344 nm, 450 nm and 476 nm) (Figure 2b). A dopant concentration of 20% Yb^3+^ and 0.5% Tm^3+^ is conventionally considered as optimal to avoid concentration quenching owing to cross-relaxation of energy between excited states in Tm^3+^-doped UCNPs.^38^ However, high concentrations of sensitizer ions (Yb^3+^) increase the effective absorption cross-section and the number of available energy transfer pathways to Tm^3+^ ions, which enable the use of higher activator concentrations while maintaining efficient upconversion emission. The luminescence decay curves confirm Tm^3+^ concentration-dependent cross–relaxation, as the lifetime of ^1^G_4_ energy level decreases with increasing concentration of Tm^3+^ ions (Supporting Information Figure S6). For the sample doped with 0.5% Tm^3+^, the luminescence lifetime of the ^1^G_4_ energy level was 625 ± 5 μs and gradually decreased to 429 ± 2 μs with increasing Tm^3+^ concentration up to 4% Tm^3+^. For 6% Tm^3+^, we observed longer luminescence lifetime of ^1^G_4_ level, which most probably results from a relatively thicker (ca. 3 nm) shell in these nanoparticles. It is important to keep a reasonable balance between concentration quenching and high luminescence emission efficiency. Although the luminescence lifetime of the ^1^G_4_ energy level was shorter when comparing 2% Tm^3+^ with the 0.5% Tm^3+^ composition, the emission intensity was the highest for 2% Tm^3+^ among all the synthesized nanoparticles.

**Figure 2:**
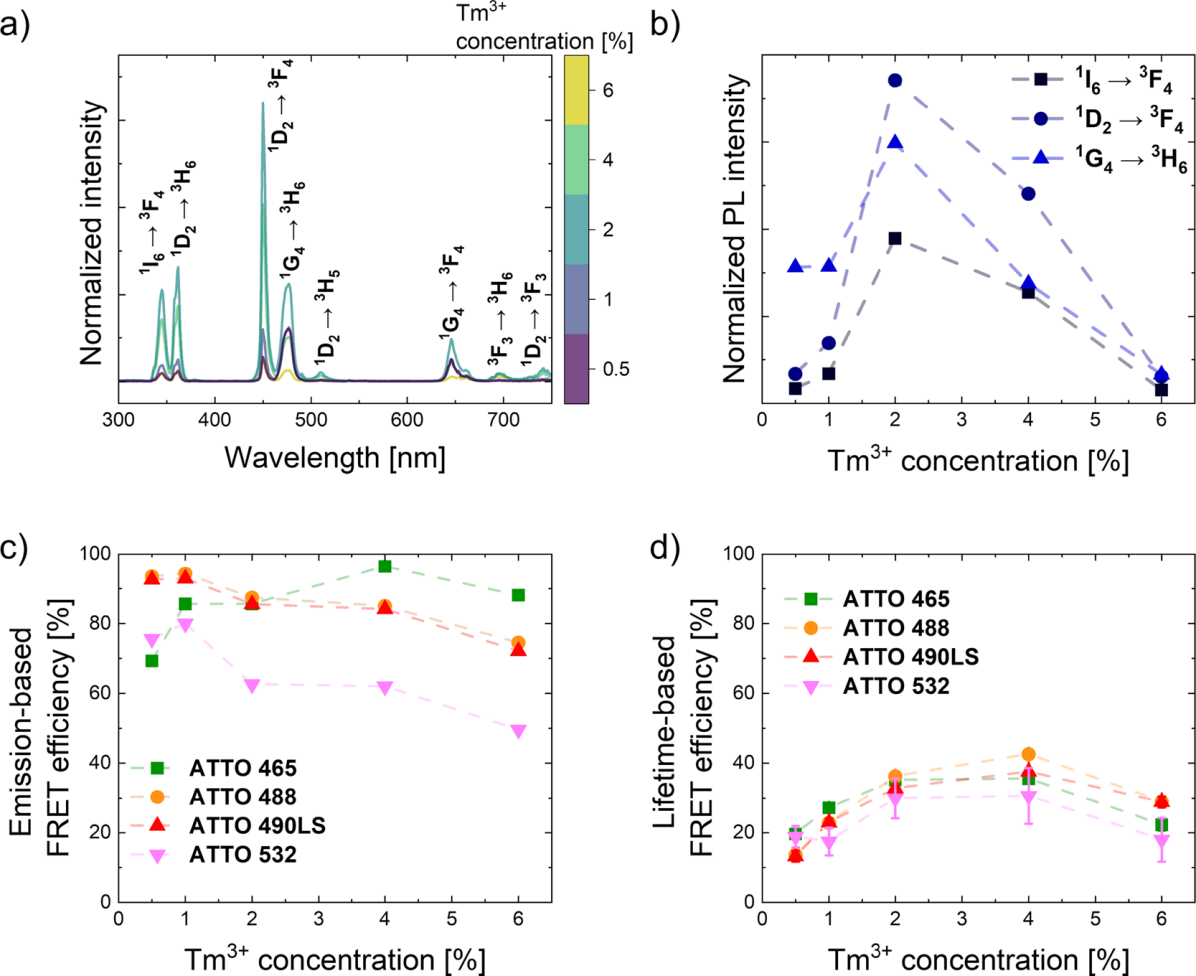
Optimization of Tm^3+^ concentration doping. (a) Emission spectra of UCNPs in chloroform, normalized to the UCNPs concentration (excitation wavelength 980 nm, power density I_P_ = 260 W/cm^2^). (b) Short wavelength emission intensities of the ^1^I_6_ → ^3^F_4_, ^1^D_2_ → ^3^F_4_, and ^1^G_4_ → ^3^H_6_ bands. Tm^3+^ concentration dependence of FRET efficiency based on (c) luminescence intensity of the donor ^1^G_4_ energy level and (d) luminescence lifetimes of the Tm^3+^: ^1^G_4_ energy level in the presence of different acceptor dyes (each 12 mg/L).

### Optimization of Tm^3+^-Based FRET

In order to evaluate the FRET phenomenon, we anchored the ATTO dyes directly onto the surface-modified UCNPs dispersed in DMF. The absorption spectra of selected ATTO dyes overlapped well with Tm^3+^: ^1^G_4_ → ^3^H_6_ emission band resulting in Förster distances equal to 2.50, 2.09, 2.42, and 1.70 nm, respectively (Supporting Information, Figures S7–S9, eqs S1–S4, and Tables S1 – S3). All ATTO dyes are equipped with a carboxy functional group, which coordinates to the lanthanide ions on the surface of UCNPs by replacing residual BF_4_^–^ groups.^39^ Initially, we investigated all the synthesized UCNPs with two different dye concentrations to quantitatively determine the optimal Tm^3+^-dopant concentration for FRET. The best UCNP composition, 50%Yb^3+^@20%Yb^3+^, 4%Tm^3+^ was then selected for more detailed FRET studies. First, the upconversion emission spectra were measured and then normalized to the integral of the Tm^3+^ IR emission band at 802 nm to reduce fluctuations of emission intensities. As expected, the donor emission decreases in the presence of acceptor dyes, which show emission maxima at 510, 540, 630, and 566 nm for ATTO 465, ATTO 488, ATTO 490LS, and ATTO 532, respectively, under continuous wave 980 nm laser excitation (Supporting Information Figure S10). The FRET efficiency was estimated using eq 1:

$$\eta =\left(1-\frac{I_{\mathrm{DA}}}{I_{D}}\right)\times 100\%$$ 1

where *I*_D_ is the integrated donor emission intensity in the absence of acceptor molecules; *I*_DA_ is the integrated donor emission intensity in the presence of acceptor molecules. Because of some residual spectral overlap of D and A, particularly for the ATTO 465 acceptor, the broad A emission profile was subtracted from the narrowband D emission to correct *I*_DA_.

No direct relationship between Tm^3+^-dopant concentration and FRET efficiency can be observed; as for smaller concentrations of ATTO 465, this efficiency increases as a function of Tm^3+^ concentration, and on the other hand, for higher dye concentrations, this relation is nonmonotonic, exhibiting alternating increasing and decreasing trends. For other dyes, with higher concentrations of Tm^3+^ ions, the energy transfer efficiency decreases (Figure 2c and Supporting Information Figure S11). This unexpected behavior originates most probably from the methodology of the signal detection. The UC-FRET spectral measurements can be highly distorted due to acceptor concentration dependent reabsorption or inner filter effect, which both are leading to overestimation of FRET efficiency. Due to the aforementioned issues, FRET efficiency was additionally calculated using luminescence lifetimes of ^1^G_4_ energy level of Tm^3+^ donor ions:

$$\eta =\left(1-\frac{\tau_{\mathrm{DA}}}{\tau_{D}}\right)\times 100\%$$ 2

where τ_D_ is the donor luminescence lifetime in the absence of acceptor molecules; τ_DA_ is the donor luminescence lifetime in the presence of acceptor molecules.

The luminescence decay times of the donor emission band decreased in the presence of an acceptor dye, resulting in the highest change from 366 ± 4 μs for 4% Tm^3+^-doped UCNPs without acceptor dye to 210 ± 2 μs for UCNPs with 12 mg/L ATTO 488 resulting in a FRET efficiency of 43% (decay curves are shown in the Supporting Information Figure S12). It was possible to obtain such a high FRET efficiency due to direct attachment of dye molecules to UCNP surface. As presented in Figure 2d, UCNPs of type NaYF_4_:50%Yb^3+^@NaYF_4_:20%Yb^3+^, 4%Tm^3+^ exhibit the highest FRET efficiencies for all 4 dyes, i.e., 36% for ATTO 465, 43% for ATTO 488, 38% for ATTO 490LS, and 29% for ATTO 532. For smaller amounts of dye (Supporting Information, Figure S13), the relationship is similar, but for ATTO 488, the highest energy transfer was observed for NaYF_4_:50%Yb^3+^@NaYF_4_:20%Yb^3+^, 2% Tm^3+^. However, this is only a negligible difference considering all the dyes; thus, we conclude that a Tm^3+^-dopant concentration of 4% in the NaYF_4_:50%Yb^3+^@NaYF_4_:20%Yb^3+^, *x*%Tm^3+^ architecture is optimal for UC-FRET.

To fully understand these results and evaluate Ln^3+^-doped UCNPs as energy donors in FRET-based assays, it is necessary to consider the specific features of the UCNP design. The dielectric core NaYF_4_ is doped with 50% of the sensitizer Yb^3+^, which increases the absorption cross-section, and then covered with a shell doped with 20% Yb^3+^ and 0.5–6% Tm^3+^ donor ions, which means ca. 630–7600 individual interactions of closest D–A may occur at the same time on the (ca. 4300 nm^2^) surface of 37 nm UCNPs. While the f–f optical transitions in the Ln^3+^ network are barely susceptible to local chemical and crystal environments, the lanthanide ions may interact over relatively long distances. Energy cross-relaxation (CR) is one of the most important factors, which not only reduces the brightness of Tm^3+^ but also shortens the luminescence lifetimes of the excited levels. On the one hand, CR is a parasitic process for the Tm^3+^ emission itself, but on the other hand, it also competes with FRET to the surface acceptors. The only way to avoid CR is to increase the distance between the Tm^3+^ ions within the host crystal but this in turn decreases the concentration of individual donor ions and reduces the number of possible D–A interactions. Overall, in order to achieve a highly efficient energy transfer to acceptors on the surface of nanoparticles, it is important to balance many factors: keep parasitic Tm^3+^-Tm^3+^ CR low enhance energy absorption, increase energy migration, and augment ETU between Yb^3+^ and Tm^3+^ ions, aiming to maximize the D–A interactions. In homogenous UCNPs, the activator ions experience volumetric activator–activator interactions that lead to concentration quenching in 3D (neighbor activators can be found from all possible directions). This Tm^3+^-Tm^3+^ CR based quenching is beneficially weakened when activators are located in a thin shell (neighbor activators can be found only in a thin 2D layer).

To gain a deeper understanding of FRET efficiency in UC-FRET systems, it is essential to distinguish between emission-based and lifetime-based FRET analyses, as none of them alone provides a complete picture of the energy transfer process. Emission-based approaches can be significantly distorted by reabsorption effects if the sample is not sufficiently diluted. In addition, donor ions located deep inside the nanoparticle volume contribute to background emission, leading to an underestimation of the FRET efficiency. Similar limitations apply to lifetime-based measurements. Donor lifetimes are not always the most reliable metric for quantifying UC-FRET, because energy migration through the Yb^3+^ network and repopulation of activator excited states may obscure direct donor–acceptor interactions.^26,32^ Nevertheless, in the Tm^3+^-doped system, the ^1^G_4_ level is populated via a three-photon process, making repopulation effects less pronounced than in previously studied Er^3+^-based UCNPs, where the corresponding green emission involves only two photons. The energy gap between Tm^3+^:^3^H_4_ and Yb^3+^:^2^F_5/2_ levels (ca. 2100 cm^–1^) is relatively large, rendering back energy transfer less efficient. Therefore, variations in the ^1^G_4_ lifetime primarily reflect changes in nonradiative energy transfer to surface-bound dyes rather than internal energy recycling. Although the repopulation of Tm^3+^ energy levels occurs from the ^2^F_5/2_ energy level of Yb^3+^, lifetime-based FRET efficiency still suggests that 4% doping of Tm^3+^ is the most efficient composition.

### Spectral and Kinetic Analysis of UC-FRET

Based on these results, we selected the 4% Tm^3+^-doped UCNPs as FRET donor to further investigate the change of donor emission spectra (Figure 3a) and lifetimes (Figure 3b) in response to a wide range of acceptor concentrations from 0.025 to 100 μM. The upconversion emission spectra clearly show that the donor emission intensity decreases in the spectral region of 430–510 nm and the emission of all acceptor dyes increases, indicating efficient UC-FRET. Figure 3a shows representative spectra for ATTO 488, while spectra of all assays are presented in the Supporting Information, Figure S14. Although the acceptor emission initially increases with concentration, higher dye concentrations lead to quenching. The quenching thresholds are 20.4 μM for ATTO 465, >1 μM for ATTO 488, 6.8 μM for ATTO 490LS, and >1 μM for ATTO 532. As ATTO 465 has the smallest molecular structure of the four dyes, more dye molecules are needed to saturate the nanoparticle surface; therefore, quenching is visible only at higher ATTO 465 concentrations. ATTO 465, ATTO 488, and ATTO 532 also show a small bathochromic shift, which may result from π–π stacking interactions and the formation of dye aggregates. At higher dye concentrations, H-like aggregates can be formed, whose regularity is constrained by the nanoparticle surface. Absorption spectra of the dye molecules (Supporting Information, Figure S15), normalized to the most intense band, show a slight increase in the hypsochromic shoulder confirming the presence of H-aggregates. However, this increase is very weak for ATTO 465, suggesting that the formation of such aggregates is less probable in this particular dye. The decrease in dye photoluminescence at higher concentrations can also be explained by homo-FRET—in this process, the excitation energy migrates between dye molecules and can be ultimately quenched by nonemissive traps.^40^ Consequently, the fluorescence of the acceptor dye decreases due to the formation of nonemissive or weakly emissive species, which introduce additional nonradiative decay pathways.^41,42^ Although acceptor emission decreases, donor emission based FRET efficiency still increases (Figure 3c,d), indicating that energy from UCNP to the dye is being transferred.

**Figure 3:**
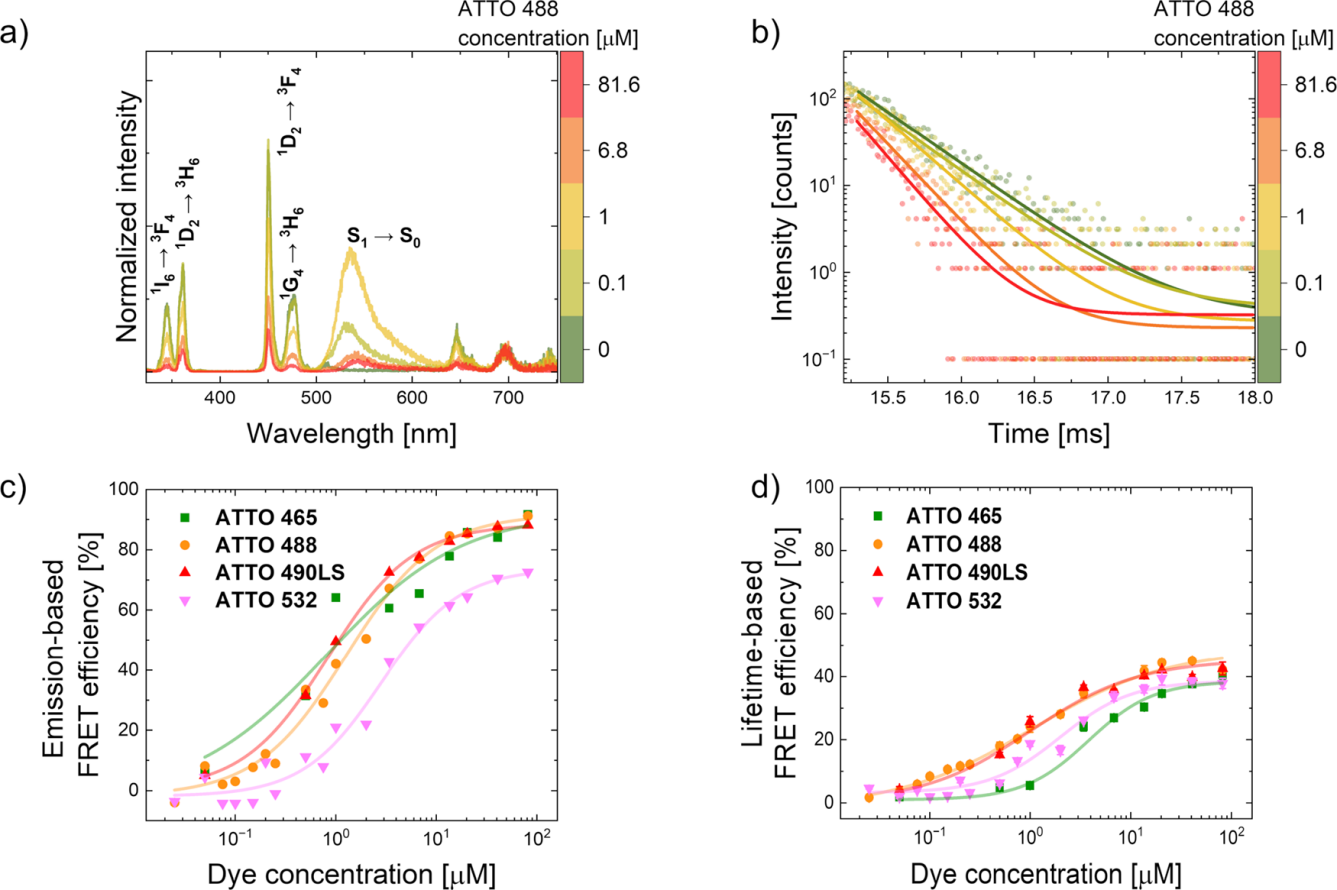
Spectral and kinetic changes of UCNPs emission (4% Tm ^3+^ in shell) in response to different acceptor dye concentrations. (a) Representative UC spectra of UCNPs in the presence of increasing ATTO 488 concentrations. (b) Representative ^1^G_4_ energy level decay curves in the presence of ATTO 488 acceptor. Dose–response curves for the four dyes based on (c) donor emission spectra and (d) donor ^1^G_4_ energy level lifetimes. These data points were fitted to a dose–response model. Full experimental FRET spectra and FRET luminescence kinetics for all the acceptor dyes are shown in the Supporting Information (Figures S14 and S16).

Conventionally, FRET efficiencies are calculated based on the donor emission or lifetimes according to eqs 1 or 2, respectively. However, these equations were developed and are strictly valid only for the molecular FRET between single donor and single acceptor molecules. UCNPs are significantly different because multiple donors (here Tm^3+^ ions) are spread at variable distances from the UCNP surface, which itself can be decorated with numerous, randomly anchored, and oriented acceptor molecules. Obviously, in such a UC-FRET assay, quantified with eq 1, the sensitivity and S/N ratio are not satisfactory, because many donor ions exist, which emit photons, but are beyond effective FRET distance.^32^ Thus, neither the reduction in donor emission nor the decay times may be influenced, which is not optimal for analyzing UC-FRET, particularly in terms of biosensing applications. This is reflected in Figure 3c,d, where the FRET efficiencies derived from eqs 1 and 2 increase, following dose–response manner with higher acceptor concentrations, and reach different saturation levels. The overestimated values from emission intensity-based analysis likely reflect a non-FRET contribution, such as the inner filter effect. Although full spectral acquisition enables appropriate corrections in response to spectral cross-talk, this approach is technically more complex and costly. The reduction of donor luminescence lifetimes is less prone to those inner filter or light scattering effects but requires costly equipment and time-consuming measurements. We may therefore conclude that quantitative UC-FRET should not rely on a single readout but combine intensity- and lifetime-based approaches with appropriate corrections.

### Optical Band-Pass Filter-Based Readout on a Microtiter Plate Reader

While typically whole emission spectra or luminescence decay times are recorded on spectrofluorometers to quantify UC-FRET, these measurements can be technically complex, expensive, and time-consuming and thus are not well suited for routine biomedical applications. A microtiter plate reader equipped with bandpass filters can measure the integrated emission bands of donor and acceptors in a cheaper and faster way. Thus, three spectral channels were defined by selecting emission intensity integrating bandpass filters: CH1 (435–485 nm), CH2 (500–550 nm) and CH3 (550–614 nm) (Figure 1d). Bhuckory et al.^29^ noted earlier that surface-attached fluorescent dyes affect the donor luminescence of UCNPs only weakly because—in line with our discussion above—only those surface lanthanide ions in FRET distance range to the dyes are quenched and they suggested the use of a so-called FRET ratio, defined as the ratio of acceptor emission to donor emission, for detection purposes. While we did see a decrease of the donor emission with increasing dye concentrations, we also found that the increase of the acceptor emission can be detected more accurately than the decrease of the donor emission. Thus, we defined the luminescence intensity ratio of the acceptor emission to the donor emission as LIR_1_. In our filter-based system, LIR_1_ is expressed as

$${\mathrm{LIR}}_{1}=\frac{\mathrm{CH}2+\mathrm{CH}3}{\mathrm{CH}1}$$ 3

With the comprehensive data set shown in Figure 4, various quantifications methods, i.e., donor intensity (CH1; rectangles), acceptor fluorescence intensity (CH2 + CH3; circles), and LIR_1_ (triangles) were compared for all acceptor dyes. It was not possible to calculate limits of detection (LOD) from the donor emissions (CH1) because the fluctuations were too high. However, calculation of the LOD based on the ratiometric readout (LIR_1_) improved the LODs about five times as compared to the calculations based on the acceptor emission alone (CH2 + CH3). The improved (i.e., lower) LOD achieved with LIR_1_ has two explanations: (1) the ratiometric readout corrects for absolute measurement fluctuations and (2) LIR_1_ combines opposing effects - the increase of acceptor emission and the decrease of donor emission for each dye concentration.

**Figure 4:**
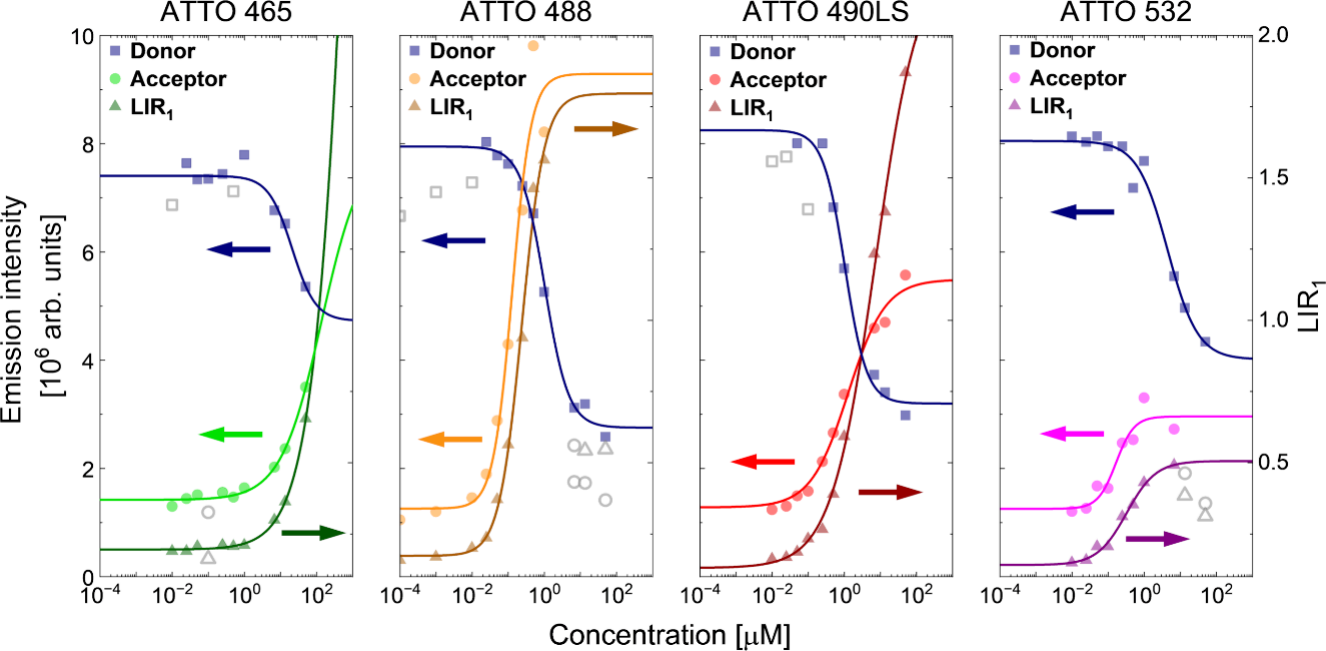
Bandpass filter-based analysis of UCNPs (4% Tm^3+^ in shell) in response to different acceptor dye concentrations. Three ways of calculating LODs (*y*_0_ + 3σ, Supporting Information, eq 5) were explored: (1) the decrease of donor (D) emission (■); (2) the increase of acceptor (A) emission (●); and (3) the ratiometric relationship A/D (LIR_1_) (▲). Empty gray datapoints were excluded from fitting; as for ATTO 488 and ATTO 532, at high dye concentrations, quenching was observed (acceptor and LIR_2_); for ATTO 465, ATTO 488, and ATTO 490LS, high fluctuations in donor emission were also noticed (donor). These points did not converge with the regression curve.

While the full spectral analysis enables correction for technical issues such as spectral overlap between D and A emission and other artifacts, such an approach is technically more complex and costly. The bandpass filter-based analysis is technically more affordable, even if it provides fewer options to correct measurement errors. The eq 1 for calculating FRET efficiencies is a ratio, thus it requires the preparation of two separate samples to measure *I*_DA_ and *I*_D_, which renders the final FRET efficiency value susceptible to systematic errors. By contrast, LIR_1_ is a self-referenced measurement because the quantification is performed on the same sample. While LIR_1_ is not directly related to physical phenomena, such as quantitate FRET efficiency or the D–A distance, the monotonic relationship between LIR_1_ and dye concentration is more suitable to perform a quantitative evaluation.

Based on LIR_1_, we calculated LODs for each combination of UCNPs and acceptor dye (Supporting Information, Figure S17). Overall, the lowest LODs were obtained for UCNPs doped with 2% of Tm^3+^ (ATTO 465: 521 nM, ATTO 488: 2.7 nM, ATTO 490LS: 24.5 nM, and ATTO 532: 34.2 nM, Supporting Information, Table S4), which was slightly surprising because originally, dopant concentration of 4% yielded the highest FRET efficiencies (Figure 2d). When compared to 4% Tm^3+^ UCNPs, the obtained LODs are approximately 2-fold lower for ATTO 465 and ATTO 488. For the other acceptor dyes, this difference is low (ATTO 532) or negligible (ATTO 490LS). However, this discrepancy can be explained by the limited validity of ‘molecular’ eqs 1 and 2 for the UC-FRET as discussed above. Furthermore, a dopant concentration of 2% yielded the highest luminescence intensity (Figure 2b), which improves the S/N ratio and might be the reason for lower LODs. Even though ATTO 465 dye seems to be the most suitable for Yb^3+^, Tm^3+^-codoped nanoparticles in terms of the calculated Förster distance (Supporting Information, Table S2), the spectral overlap between donor and acceptor emission might be the reason for suboptimal LODs.^16^

### Multiplexed UC-FRET

For multiplexed UC-FRET analysis aimed to distinguish various acceptor dyes anchored to one type of UCNP (4% Tm^3+^ in shell), we defined three spectral channels as depicted in Figure 1d. In the spectral analysis, a certain spectral range was integrated by the software, while on the microtiter plate reader, physical bandpass filters integrated photons in given spectral ranges within the UC well-plate reader. While CH1 (435–485 nm) integrated the blue donor emission, the CH2 (500–550 nm) and CH3 (550–614 nm) integrated the fluorescence intensities in the spectral gap between the two emission bands of Tm^3+^ ions, aiming to split the available range into 2 halves and let us define a ratiometric measure:

$${\mathrm{LIR}}_{2}=\frac{\mathrm{CH}2}{\mathrm{CH}2+\mathrm{CH}3}$$ 4

Based on the LIR_2_ values, a simple threshold classification for four acceptor dyes was proposed:

$${\mathrm{Threshold}}_{i/j}=\frac{\mu_{{\mathrm{dye}}_{i}}+\mu_{{\mathrm{dye}}_{j}}}{2}$$ 5

where μ_dye_*i*__ is the mean value of LIR_2_ distribution of dye *i* and μ_dye_*j*__ is the mean value of LIR_2_ distribution of dye *j*, where *i* and *j* denote the dyes, whose LIR_2_ values are most similar. These calculated thresholds are marked on Figure 5a,b, as thin, gray, dotted horizontal lines.

**Figure 5:**
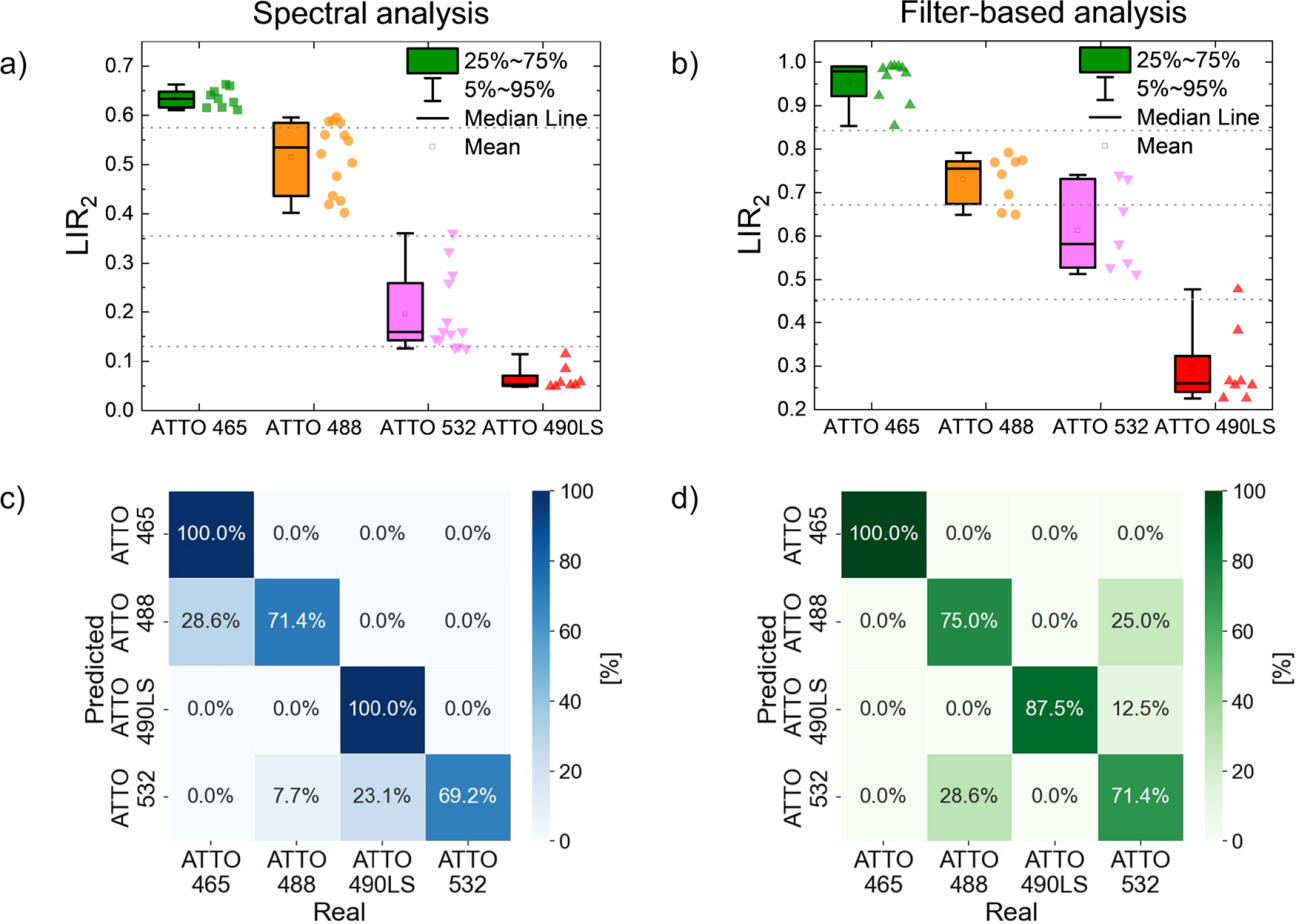
Analysis of the UC-FRET multiplexing. Analysis of 4 channel UC-FRET using spectral (FLS 1000 spectrofluorometer) and bandpass filter (Labrox well-plate reader) readout. Acceptor concentration-dependent LIR_2_ values distribution calculated for (a) emission spectra and (b) two bandpass integral emission intensities ratio; gray dotted line represents thresholds. The corresponding confusion matrices are presented for (c) emission spectra and (d) filter-based measurements, respectively. The numbers on the diagonal represent the percentage of correctly predicted dyes.

It is obvious that LIR_2_ is different for each of the acceptor dyes. Although ATTO 465 and ATTO 490LS seem to be well separated from other dyes by this threshold, one can notice that the LIR_2_ values of ATTO 488 overlap with those of ATTO 465. For ATTO 532, LIR_2_ values overlap with ATTO 488 and ATTO 490LS values (Figure 5a). The spectral analysis approach, however, required a careful correction of the acceptor emission overlapping with the donor channel. Thus, we also used the two optical bandpass filters CH2 and CH3 on the microtiter plate reader. The integrated intensity in CH2 was related to the integrated intensity of all of the acceptor channels (CH2 + CH3). Here, a similar behavior can be observed. However, LIR_2_ values are more widely distributed for ATTO 488 and ATTO 532 resulting in their higher overlap (Figure 5b). For the multiplexed detection experiments, only one dye was present at a given time, and no mixtures of dyes were measured. The spectral analysis and filter-based analysis yielded similar classification results. The distribution of LIR_2_ values may result from background fluctuations and readout noise, which were observed especially at low dye concentrations. At very high acceptor concentrations, it was difficult to differentiate LIR_2_ values due to the bathochromic shift of the acceptor emission, particularly for ATTO 488 and ATTO 532 acceptors.

Despite these challenges, the simple threshold-based method achieved classification accuracies of 81.8% for the spectral analysis and 84.8% for the filter-based analysis that demonstrates the potential of the LIR_2_ figure of merit as a simple yet effective and reliable discriminative metric in multiplexed FRET analysis (Figure 5c,d). The confusion matrices reveal that most errors occur for ATTO 532 dye since its absorption band has the lowest spectral overlap with the Tm^3+^ emission and its brightness and S/N ratio are the lowest among all the analyzed dyes. The best accuracy is observed for ATTO 465, due to its strong emission signal. While this is a very simple demonstration of multiplexing technique, for more advanced biorelated applications, a cross-talk correction could be used, which could reduce LODs and allow an even better dye distinction.^43^ These multiplexing capabilities may not be sufficient yet to analyze signals in mixed, homogeneous assays; however, they can help in digital assays to distinguish numerous individual and specifically biofunctionalized donor nanoparticles that are exposed to complex biosamples, and then massively analyzed using wide-field UC imaging.^44,45^

## Conclusions and Outlook

In summary, we evaluated the impact of the concentration of Tm^3+^ ions within the optimized NaYF_4_:50%Yb^3+^@NaYF_4_:20%Yb^3+^, *x*% Tm^3+^ core@shell nanocrystals architecture on emission intensity and UC-FRET efficiency. The 2% Tm^3+^-doped sample was found to be the brightest, whereas a higher Tm^3+^ doping concentration of 4% was the most beneficial for the UC-FRET efficiency. According to molecular FRET theory, the FRET efficiency can be quantified either in the spectral or time domain. However, we have found that the evaluation of FRET based solely on emission spectra can be significantly biased and overestimated due to the spectral overlap between D and A fluorescence. The use of Yb^3+^/Tm^3+^ codoped UCNP donors resulted in high lifetime-based FRET efficiencies of more than 40%. These results however show complexity behind UC-FRET mechanism and available methods serving to evaluate its efficiency. Both intensity and lifetime clearly give very different results, and FRET efficiency analysis must be performed using whole set of data for deep understanding of the process itself. From the perspective of point-of-care diagnostics, it is clear that ratiometric approaches which provide the lowest LODs are technically more simple, cost-effective, and affordable. Furthermore, the broad spectral separation between the blue and red emission bands of Tm^3+^ ions (ca. 170 nm compared to ca. 100 nm of Er^3+^ donor ions) enabled multiplexed detection of four different fluorescent dyes using a single donor species. Our multiplexing analysis based solely on the integrated emission intensities collected with two bandpass filters, achieved an accuracy of 84.8%, highlighting the potential of this system for advanced optical sensing applications. The potential to distinguish four analytes on simple detection instruments with a minimum technical requirement of only one excitation wavelength and two/three emission filters should tremendously simplify the readout of UC-FRET bioassays. Thus, this simple ratiometric detection scheme holds great advantage for multiplexed, fast, sensitive, and wash-free point-of-care (POC) bioassays.^46^

## Experimental Section

### Materials

Yttrium oxide (99.99%), ytterbium oxide (99.99%), and thulium oxide (99.99%) were purchased from Alfa Aesar. Dry DMF (99.8%), nitrosonium tetrafluoroborate (95%), oleic acid (OA, 90%), and 1-octadecene (ODE, 90%) were purchased from Sigma-Aldrich. Acetic acid (99.5–99.9%), ethanol (96%), *n*-hexane (95%), methanol (99.8%), ammonium fluoride (98%), toluene (99.5%), acetonitrile (99.9–99.99%), and sodium hydroxide (98.8%) were purchased from Avantor Performance Materials S.A. (Poland). Carboxy derivatives of ATTO 465, ATTO 488, ATTO 490LS, and ATTO 532 dyes were purchased from ATTO-TEC company (Germany).

### Synthesis

#### Precursors for Synthesis

Stoichiometric amounts of lanthanide oxides were mixed with 50% aqueous acetic acid. A mixture was transferred to a separate Teflon vessel and heated to 200 °C for 120 min. Then, the residual acetic acid and water were evaporated, and the core precursor was left to dry for 12 h on a hot plate at 140 °C.

#### Core Nanoparticles

β-NaYF_4_:50%Yb^3+^ core nanoparticles were synthesized using a thermal decomposition method. In a typical procedure, oleic acid (30 mL) and 1-octadecene (75 mL) were added to a flask containing 2.5 mmol of yttrium and 2.5 mmol of ytterbium acetates serving as precursor. The mixture was stirred and heated to 140 °C for 30 min under vacuum to remove oxygen and residual water. Then, the mixture was cooled to 70 °C, and NH_4_F (20 mmol) and NaOH (12.5 mmol) in methanol were added. Subsequently, the solution was stirred for 15 min. Next, the temperature was increased to 140 °C, and the mixture was kept under these conditions to evaporate methanol. The solution was then quickly heated to 300 °C under nitrogen atmosphere and maintained at this temperature for 1 h. Afterward, the mixture was cooled down to 70 °C, and the nanoparticles were precipitated in an excess of ethanol and centrifuged at 10,000 rpm for 10 min. The supernatant was removed, and the nanoparticles were washed with *n*-hexane, precipitated again by addition of ethanol, and centrifuged at 14,000 rpm for 10 min. Finally, the supernatant was removed, and the as-prepared nanoparticles were dispersed in 10 mL of chloroform.

#### Core@Shell Nanoparticles

The core@shell nanoparticles were synthesized via the thermal decomposition of trifluoroacetates. First, to a flask containing 1.25 mmol of the shell precursor (yttrium, ytterbium, and thulium trifluoroacetates in proper proportions), sodium trifluoroacetate (2.5 mmol), oleic acid (20 mL), and 1-octadecene (20 mL) were added. Then, the mixture was heated to 120 °C under vacuum and kept under these conditions until a clear solution was formed. Subsequently, the solution was cooled down to 60 °C, and 5 mL of β-NaYF_4_:50%Yb^3+^ core nanoparticles colloidal solution in chloroform was added under a nitrogen atmosphere. Afterward, chloroform was evaporated. First, the temperature was increased to 80 °C, and the flask was opened for 30 min. Subsequently, the temperature was raised to 110 °C, and the solution was kept under vacuum for 15 min. Then, the solution was quickly heated to 300 °C under a nitrogen atmosphere and maintained under these conditions for 1 h. Finally, the solution was cooled to 70 °C, and the nanoparticles were precipitated as described above.

#### Material Characterization

Powder diffraction data were collected on a X’Pert PRO X-ray diffractometer with a PIXcel ultrafast line detector, a focusing mirror, and Soller slits for Cu Kα radiation. The measurements were done in a Bragg–Brentano geometry in the 10°–90° 2θ range. The powder X-ray diffraction (XRD) patterns were assigned using the ICSD database. Transmission electron microscope images were taken with a Philips CM20 SuperTwin at 160 kV.

#### Surface Modification of UCNPs

As-synthesized nanoparticles were next functionalized using procedure with NOBF_4_.^47,48^ First, the nanoparticles were centrifuged for 30 min at 16,900 rpm. Next the supernatant was removed, 500 μL of *n*-hexane was added, and nanoparticles were dispersed. Then, 400 μL of acetonitrile and 100 μL 0.16 M NOBF_4_ in acetonitrile were added. Nanoparticles were mixed in order to transfer all of them to a lower acetonitrile layer. The hexane layer was removed, and nanoparticles were precipitated with 500 μL of toluene. Afterward, they were vortexed and centrifuged for 30 min at 16,900 rpm. Subsequently, the supernatant was removed, and nanoparticles were dispersed in 100 μL of dry dimethylformamide (DMF).

#### Attachment of Dye Molecules

To 5 μL of nanoparticles dispersed in DMF, different volumes of dye solutions in DMF (concentration 0.63 mg/mL) were added and incubated for 10 min. The total volume of DMF was then adjusted to 500 μL, obtaining a dye concentration range from 0.025 to 81.6 μM. Samples for measurements using Labrox microtiter plate reader were prepared in a similar way. For each sample, three measurements were performed by placing 50 μL of solution in each well. The concentration ranges were adjusted to obtain enough experimental points for logistic curve fitting: from 0.01 to 50 μM (ATTO 465, ATTO 490LS and ATTO 532) and from 0.1 nM to 50 μM for ATTO 488.

#### Absorption Measurements

Absorption spectra of dyes were acquired using a Cary Varian 5E UV–vis–NIR.

#### Emission and Lifetime Measurements

Emission and lifetime measurements were conducted with FLS 1000 Edinburgh Instruments equipped with a 980 nm photoexcitation wavelength laser diode (Spectra-Laser). The luminescence lifetimes were obtained with utilization of FLS 1000 with a R928P photomultiplier tube detector (Hamamatsu) under excitation with a TTL-modulated 980 nm CW laser diode. For spectral analysis donor (and donor in the presence of acceptor), emission intensity was calculated as an integral of ^1^G_4_ → ^3^H_6_ transition in the range of 460–490 nm.

#### Labrox Reader

The upconversion emission of Tm^3+^-doped UCNPs was measured on an upconversion microtiter plate reader (UPCON, Labrox, Turku, Finland) equipped with a 976 nm continuous wave (CW) laser, integrated excitation filter (976/30), and a short-pass dichroic mirror (D900 nm). Bandpass filters for the emission light were selected to define the three detection channels CH1–3: 460/50 (Chroma) for CH1, 525/50 (Chroma) for CH2, and 582/64 (Semrock) for CH3. In each well, 4 × 4 points were raster-scanned in 700-μm steps and a signal integration time of 500 ms. A trimmed mean for each well was calculated by removing the lowest and highest 5% of the data points (middle 90th quantile). The plotted averages and standard deviations were determined from three independent wells. The data was fitted by a four-parameter logistic function. LODs were obtained by adding three times the standard deviation of the blank to the baseline of the regression curve.

## Supplementary Material



## Notes

TEM and XRD of nanocrystals; lifetime analysis of oleic-capped UCNPs; Förster distance calculations; dye absorption spectra; emission spectra of UCNPs conjugated with dye molecules; luminescence kinetics of UCNPs conjugated with dye molecules; emission-based and lifetime-based FRET efficiency; LIR_1_ dose–response curves; limits of detection; and calculation of number of dye molecules per one nanocrystals and photostability test of UCNPs conjugated with dye molecules (PDF)

## References

[1] Theodor
Förster (1946). Energiewanderung
und Fluoreszenz. Naturwissenschaften.

[2] Medintz, I. L. ; Hildebrandt, N. FRET - Förster Resonance Energy Transfer: From Theory to Applications; Wiley-VCH Verlag GmbH, 2014.

[3] Yu X., Dou L., Ma M., Yu W., Wen K., Ke Y., Shen J., Zhang S., Wang Z. (2022). Construction of Label-Free
FRET Immunoassays Using Three Antibody Fragments to Insight into the
Structural Basis of Sensitivity Difference. Sens. Actuators, B.

[4] Strianese M., De Martino F., Pavone V., Lombardi A., Canters G. W., Pellecchia C. (2010). A FRET-Based Biosensor for NO Detection. J. Inorg. Biochem..

[5] Yu Y., Li W., Gu X., Yang X., Han Y., Ma Y., Wang Z., Zhang J. (2022). Inhibition of CRISPR-Cas12a Trans-Cleavage
by Lead (II)-Induced G-Quadruplex and Its Analytical Application. Food Chem..

[6] Chu-Mong K., Thammakhet C., Thavarungkul P., Kanatharana P., Buranachai C. (2016). A FRET Based
Aptasensor Coupled with Non-Enzymatic
Signal Amplification for Mercury (II) Ion Detection. Talanta.

[7] Kaur A., Ellison M., Dhakal S. (2021). MASH-FRET: A Simplified
Approach
for Single-Molecule Multiplexing Using FRET. Anal. Chem..

[8] Qiu X., Hildebrandt N. (2019). A Clinical
Role for Förster Resonance Energy
Transfer in Molecular Diagnostics of Disease. Expert Review of Molecular Diagnostics. Taylor and Francis Ltd..

[9] Algar W. R., Hildebrandt N., Vogel S. S., Medintz I. L. (2019). FRET as a Biomolecular
Research Tool — Understanding Its Potential While Avoiding
Pitfalls. Nat. Methods.

[10] Su R., Francés-Soriano L., Diriwari P. I., Munir M., Haye L., So̷rensen T. J., Díaz S. A., Medintz I. L., Hildebrandt N. (2025). FRET Materials
for Biosensing and
Bioimaging. Chem. Rev..

[11] Zhao M., Wang J., Lei Z., Lu L., Wang S., Zhang H., Li B., Zhang F. (2021). NIR-II pH
Sensor with
a FRET Adjustable Transition Point for In Situ Dynamic Tumor Microenvironment
Visualization. Angew. Chem..

[12] Song Y., Madahar V., Liao J. (2011). Development
of FRET Assay into Quantitative
and High-Throughput Screening Technology Platforms for Protein-Protein
Interactions. Ann. Biomed. Eng..

[13] Rainey K. H., Patterson G. H. (2019). Photoswitching FRET to Monitor Protein-Protein Interactions. Proc. Natl. Acad. Sci. U. S. A..

[14] Higuera-Rodriguez R.
A., De Pascali M. C., Aziz M., Sattler M., Rant U., Kaiser W. (2023). Kinetic FRET
Assay to Measure Binding-Induced Conformational
Changes of Nucleic Acids. ACS Sens..

[15] Maslov I., Volkov O., Khorn P., Orekhov P., Gusach A., Kuzmichev P., Gerasimov A., Luginina A., Coucke Q., Bogorodskiy A., Gordeliy V., Wanninger S., Barth A., Mishin A., Hofkens J., Cherezov V., Gensch T., Hendrix J., Borshchevskiy V. (2023). Sub-Millisecond
Conformational Dynamics of the A2A Adenosine Receptor Revealed by
Single-Molecule FRET. Commun. Biol..

[16] Grant D. M., Zhang W., McGhee E. J., Bunney T. D., Talbot C. B., Kumar S., Munro I., Dunsby C., Neil M. A. A., Katan M., French P. M. W. (2008). Multiplexed FRET
to Image Multiple
Signaling Events in Live Cells. Biophys. J..

[17] Lerner E., Cordes T., Ingargiola A., Alhadid Y., Chung S. Y., Michalet X., Weiss S. (2018). Toward Dynamic Structural Biology:
Two Decades of Single-Molecule Förster Resonance Energy Transfer. Science.

[18] Panwar J., Merten C. A. (2023). Fluorescence Crosstalk Reduction by Modulated Excitation-Synchronous
Acquisition for Multispectral Analysis in High-Throughput Droplet
Microfluidics. Lab Chip.

[19] Miller R. C., Aplin C. P., Kay T. M., Leighton R., Libal C., Simonet R., Cembran A., Heikal A. A., Boersma A. J., Sheets E. D. (2020). FRET Analysis of
Ionic Strength Sensors in the Hofmeister
Series of Salt Solutions Using Fluorescence Lifetime Measurements. J. Phys. Chem. B.

[20] Leavesley S. J., Rich T. C. (2016). Overcoming Limitations
of FRET Measurements. Cytom. A.

[21] Liu F. T., Jiang P. F., Wang Y. P., Zhao B. X., Lin Z. M. (2024). A Ratiometric
Fluorescent Probe Based on the FRET Platform for the Detection of
Sulfur Dioxide Derivatives and Viscosity. Anal.
Chim. Acta.

[22] Naczynski D. J., Tan M. C., Zevon M., Wall B., Kohl J., Kulesa A., Chen S., Roth C. M., Riman R. E., Moghe P. V. (2013). Rare-Earth-Doped Biological Composites as in Vivo Shortwave
Infrared Reporters. Nat. Commun..

[23] Gnach A., Lipinski T., Bednarkiewicz A., Rybka J., Capobianco J. A. (2015). Upconverting
Nanoparticles: Assessing the Toxicity. Chem.
Soc. Rev..

[24] Chen G., Qiu H., Prasad P. N., Chen X. (2014). Upconversion Nanoparticles: Design,
Nanochemistry, and Applications in Theranostics. Chem. Rev..

[25] Tan G. R., Wang M., Hsu C. Y., Chen N., Zhang Y. (2016). Small Upconverting
Fluorescent Nanoparticles for Biosensing and Bioimaging. Adv. Opt. Mater..

[26] Kotulska A. M., Pilch-Wróbel A., Lahtinen S., Soukka T., Bednarkiewicz A. (2022). Upconversion
FRET Quantitation: The Role of Donor Photoexcitation Mode and Compositional
Architecture on the Decay and Intensity Based Responses. Light: Sci. Appl..

[27] Casillas-Rubio A., Hamraoui K., Mendez-Gonzalez D., Laurenti M., Rubio-Retama J., Calderón O. G., Melle S. (2025). Influence of Excitation Pulse Duration
on the Efficiency of Upconversion Nanoparticle-Based FRET. Nanoscale.

[28] Bhuckory S., Hemmer E., Wu Y. T., Yahia-Ammar A., Vetrone F., Hildebrandt N. (2017). Core or Shell?
Er3+ FRET Donors in
Upconversion Nanoparticles. Eur. J. Inorg. Chem..

[29] Bhuckory S., Lahtinen S., Höysniemi N., Guo J., Qiu X., Soukka T., Hildebrandt N. (2023). Understanding FRET in Upconversion
Nanoparticle Nucleic Acid Biosensors. Nano Lett..

[30] Muhr V., Würth C., Kraft M., Buchner M., Baeumner A. J., Resch-Genger U., Hirsch T. (2017). Particle-Size-Dependent Förster
Resonance Energy Transfer from Upconversion Nanoparticles to Organic
Dyes. Anal. Chem..

[31] Pini F., Francés-Soriano L., Andrigo V., Natile M. M., Hildebrandt N. (2023). Optimizing
Upconversion Nanoparticles for FRET Biosensing. ACS Nano.

[32] Pilch-Wrobel A., Kotulska A. M., Lahtinen S., Soukka T., Bednarkiewicz A. (2022). Engineering
the Compositional Architecture of Core-Shell Upconverting Lanthanide-Doped
Nanoparticles for Optimal Luminescent Donor in Resonance Energy Transfer:
The Effects of Energy Migration and Storage. Small.

[33] Bednarkiewicz A., Nyk M., Samoc M., Strek W. (2010). Up-Conversion FRET from Er3+/Yb3+:NaYF4
Nanophosphor to CdSe Quantum Dots. J. Phys.
Chem. C.

[34] Siefe C., Mehlenbacher R. D., Peng C. S., Zhang Y., Fischer S., Lay A., McLellan C. A., Alivisatos A. P., Chu S., Dionne J. A. (2019). Sub-20
Nm Core-Shell-Shell Nanoparticles for Bright Upconversion and Enhanced
Förster Resonant Energy Transfer. J.
Am. Chem. Soc..

[35] Nsubuga A., Fayad N., Pini F., Natile M. M., Hildebrandt N. (2025). Small Upconversion-Ruthenium
Nanohybrids for Cancer Theranostics. Nanoscale.

[36] Nsubuga A., Morice K., Fayad N., Pini F., Josserand V., Le Guével X., Alhabi A., Henry M., Puchán
Sánchez D., Plassais N., Josse P., Boixel J., Blanchard P., Cabanetos C., Hildebrandt N. (2025). Sub 20 Nm
Upconversion Photosensitizers for Near-Infrared Photodynamic Theranostics. Adv. Funct. Mater..

[37] Corbella
Bagot C., Rappeport E., Das A., Ba Tis T., Park W. (2022). True FRET-Based Sensing of pH via Separation of FRET and Photon Reabsorption. Adv. Opt. Mater..

[38] Misiak M., Prorok K., Cichy B., Bednarkiewicz A., Strȩk W. (2013). Thulium Concentration Quenching in the Up-Converting
α-Tm 3+/Yb3+ NaYF4 Colloidal Nanocrystals. Opt. Mater. (Amst).

[39] Andresen E., Resch-Genger U., Schäferling M. (2019). Surface Modifications for Photon-Upconversion-Based
Energy-Transfer Nanoprobes. Langmuir.

[40] Chen C., Corry B., Huang L., Hildebrandt N. (2019). FRET-Modulated
Multihybrid Nanoparticles for Brightness-Equalized Single-Wavelength
Barcoding. J. Am. Chem. Soc..

[41] Lu L., Jones R. M., McBranch D., Whitten D. (2002). Surface-Enhanced Superquenching
of Cyanine Dyes as J-Aggregates on Laponite Clay Nanoparticles. Langmuir.

[42] Kometani N., Nakajima H., Asami K., Yonezawa Y., Kajimoto O. (2000). Luminescence
Properties of the Mixed J-Aggregate of Two Kinds of Cyanine Dyes in
Layer-by-Layer Alternate Assemblies. J. Phys.
Chem. B.

[43] Geißler D., Stufler S., Löhmannsröben H. G., Hildebrandt N. (2013). Six-Color Time-Resolved Förster Resonance Energy
Transfer for Ultrasensitive Multiplexed Biosensing. J. Am. Chem. Soc..

[44] Mickert M. J., Farka Z., Kostiv U., Hlaváček A., Horák D., Skládal P., Gorris H. H. (2019). Measurement of Sub-Femtomolar
Concentrations of Prostate-Specific Antigen through Single-Molecule
Counting with an Upconversion-Linked Immunosorbent Assay. Anal. Chem..

[45] Brandmeier J. C., Jurga N., Grzyb T., Hlaváček A., Obořilová R., Skládal P., Farka Z., Gorris H. H. (2023). Digital and Analog
Detection of SARS-CoV-2
Nucleocapsid Protein via an Upconversion-Linked Immunosorbent Assay. Anal. Chem..

[46] Farka Z., Brandmeier J. C., Mickert M. J., Pastucha M., Lacina K., Skládal P., Soukka T., Gorris H. H. (2024). Nanoparticle-Based
Bioaffinity Assays: From the Research Laboratory to the Market. Adv. Mater..

[47] Sedlmeier A., Gorris H. H. (2015). Surface Modification and Characterization of Photon-Upconverting
Nanoparticles for Bioanalytical Applications. Chem. Soc. Rev..

[48] Dong A., Ye X., Chen J., Kang Y., Gordon T., Kikkawa J. M., Murray C. B. (2011). A Generalized
Ligand-Exchange Strategy Enabling Sequential
Surface Functionalization of Colloidal Nanocrystals. J. Am. Chem. Soc..

