# Supplementary material for: Multicolor Upconversion Förster Resonant Energy Transfer Using Optimized Yb@YbTm Core@Shell Nanoparticles
Source: ACS Nano. 2025 Nov 24;19(48):41110–20. doi: 10.1021/acsnano.5c13869 (PMC12874630; doi:10.1021/acsnano.5c13869)
Supplement: Supplementary file 1 [file nn5c13869_si_001.pdf]

Supporting information

Multicolor Upconversion Förster Resonant Energy Transfer Using Optimized Yb@YbTm Core@Shell Nanoparticles

Grzegorz Bękarski[1], Katarzyna Prorok[1], František Štětina[2], Małgorzata Misiak[1], Hans H. Gorris[2], Artur Bednarkiewicz[1]*

[1]Institute of Low Temperature and Structure Research, Polish Academy of Sciences, ul. Okólna 2, 50-422 Wroclaw, Poland

[2]Department of Biochemistry, Faculty of Science, Masaryk University, Kamenice 5, 625 00 Brno, Czech Republic

*corresponding author, e-mail: a.bednarkiewicz@intibs.pl

Table of contents

1. XRD patterns of core nanocrystals 3

2. TEM images of synthetized core nanoparticles 4

3. XRD patterns of core@shell nanocrystals 5

4. TEM images of synthesized core@shell nanocrystals 6

5. Analysis of oleic-capped UCNPs dispersed in chloroform luminescence kinetics 7

5.1. Luminescence decay curves 7

5.2. Luminescence lifetimes of nanoparticles dispersed in chloroform 7

6. Förster distance 8

6.1 Dye structures 8

6.2 Calculation of Förster distance 8

6.3 Donor quantum yield discussion 11

7. Dye absorption spectra 12

8. Experimental data for optimization of Tm^{3+} concentration – emission spectra 13

9. Emission-based FRET efficiency at 4 mg/L dye concentration 14

10. Experimental data for optimization of Tm^{3+} concentration – luminescence lifetimes 15

11. Lifetime-based FRET efficiency at 4 mg/L dye concentration 16

12. Analysis of donor emission in the presence of acceptor 16

13. Investigation of dye aggregates formation 17

14. Analysis of luminescence lifetimes of $^1G_4$ energy level in the presence of acceptor 18

15. Estimation of LOD 19

15.1 LIR_1 dose – response curves 19

15.2 Summary of LODs 20

16. **Calculation of dye molecules per nanocrystal** 20
17. **Photostability of UCNPs conjugated with dye molecules** 23
18. **References** 24

1. XRD patterns of core nanocrystals

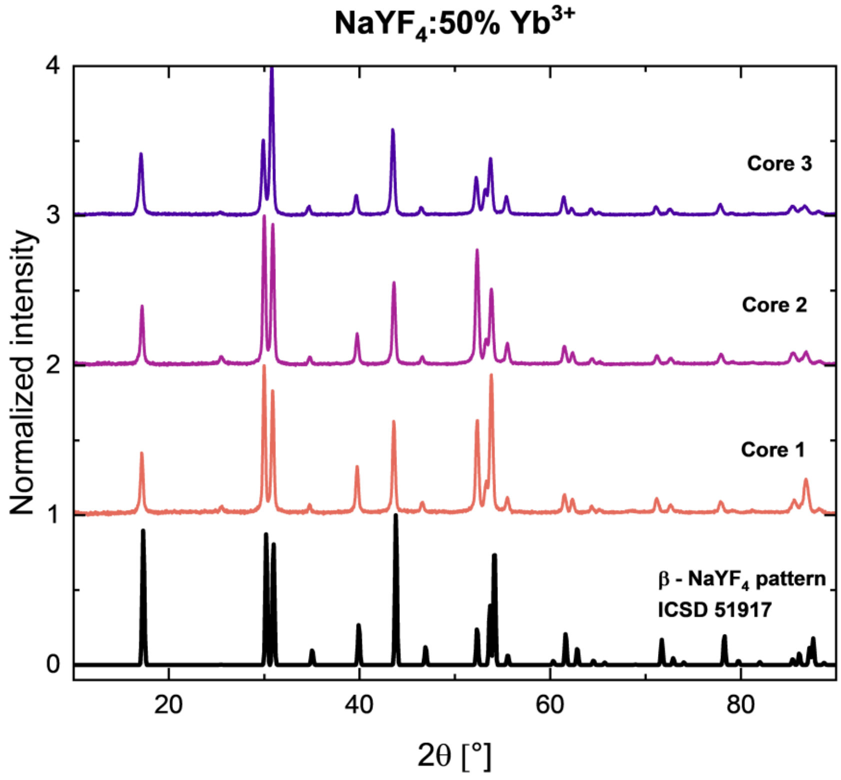

Figure S1. XRD patterns of core $NaYF_4$:50%Yb^{3+}.

2. TEM images of synthetized core nanoparticles

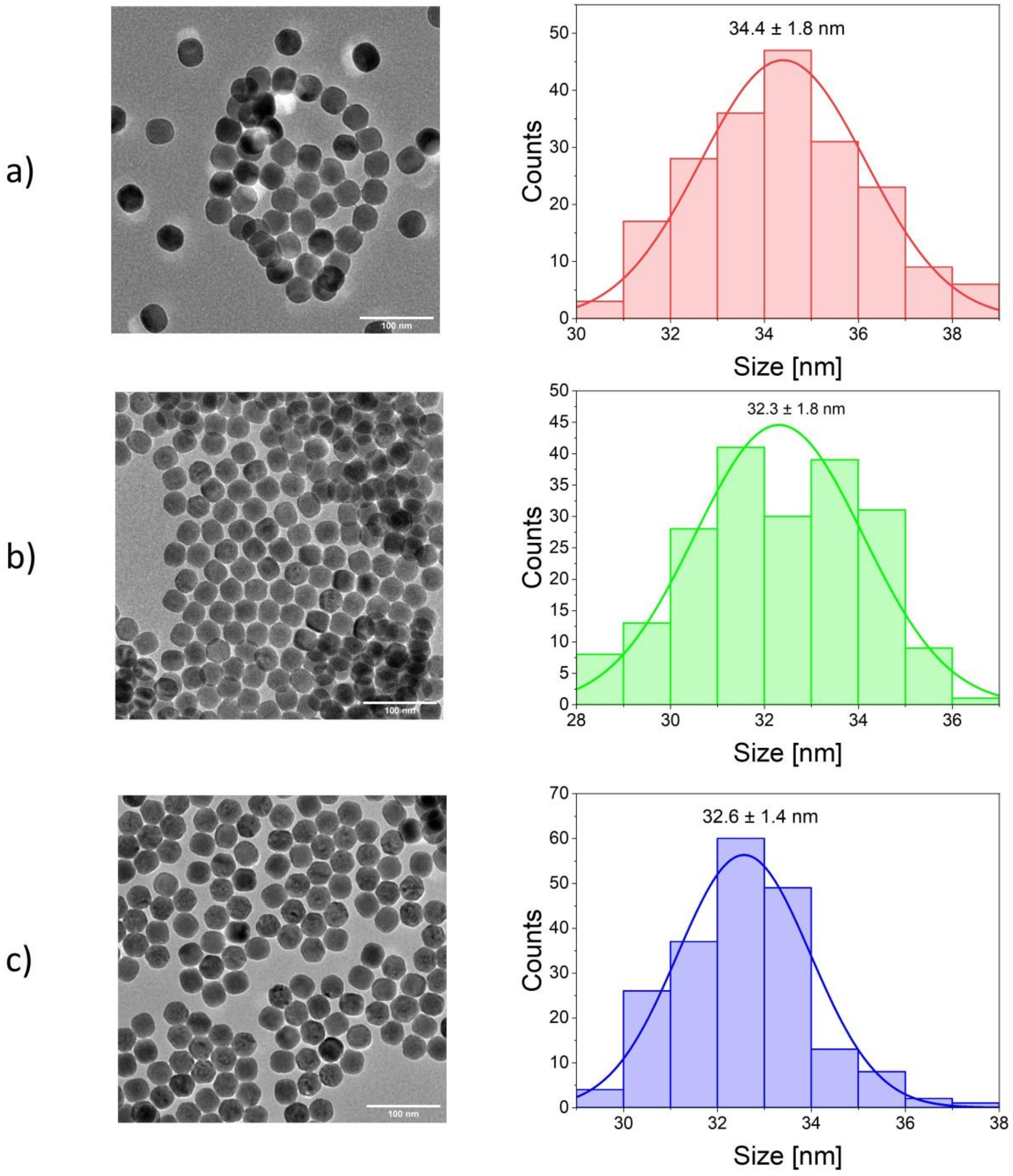

Figure S2. TEM images and corresponding diameter histograms (a, b and c – different batches of $NaYF_4$:50%Yb^{3+} core nanocrystals).

3. XRD patterns of core@shell nanocrystals

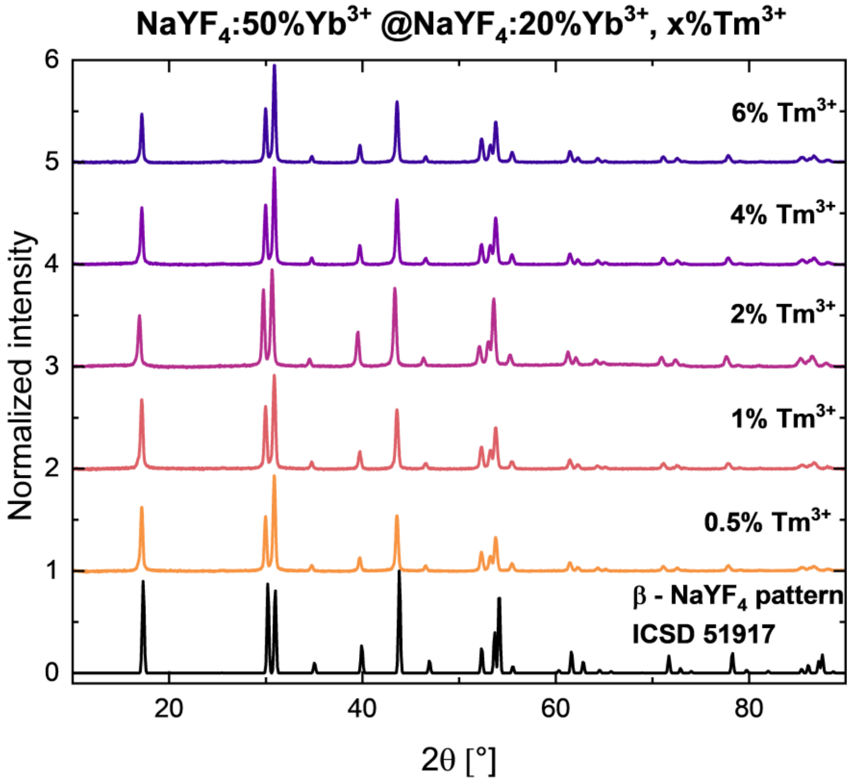

Figure S3. XRD patterns of core@shell synthesized $NaYF_4$:50%Yb^{3+}@$NaYF_4$:20%Yb^{3+}, x%Tm^{3+} nanocrystals.

4. TEM images of synthesized core@shell nanocrystals

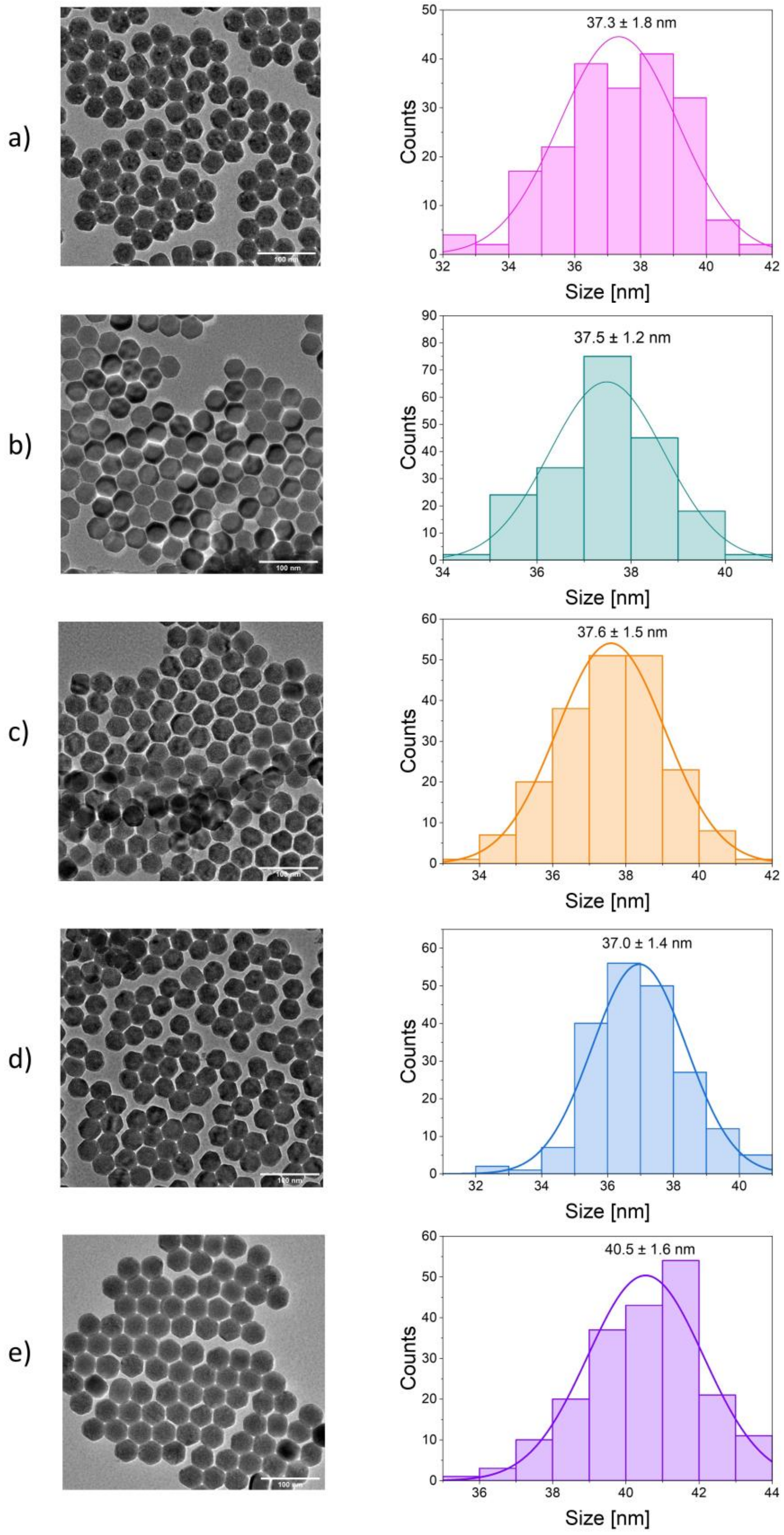

Figure S4. TEM images of $NaYF_4$:50%Yb^{3+}@$NaYF_4$:20%Yb^{3+}, x%Tm^{3+} core@shell nanocrystals where x is equal to a) 0.5% Tm^{3+}, b) 1% Tm^{3+}, c) 2% Tm^{3+}, d) 4% Tm^{3+}, e) 6% Tm^{3+}.

For estimating shell thickness of each core@shell nanocrystal composition it is important to state that 0.5% Tm^{3+} and 6% Tm^{3+} were synthesized from 34.4 nm core (Figure S2a); 1% Tm^{3+} and 2% Tm^{3+} were synthesized from 32.3 nm core (Figure S2b); 4% Tm^{3+} were synthesized from 32.6 nm core (Figure S2c).

5. Analysis of oleic-capped UCNPs dispersed in chloroform luminescence kinetics

5.1. Luminescence decay curves

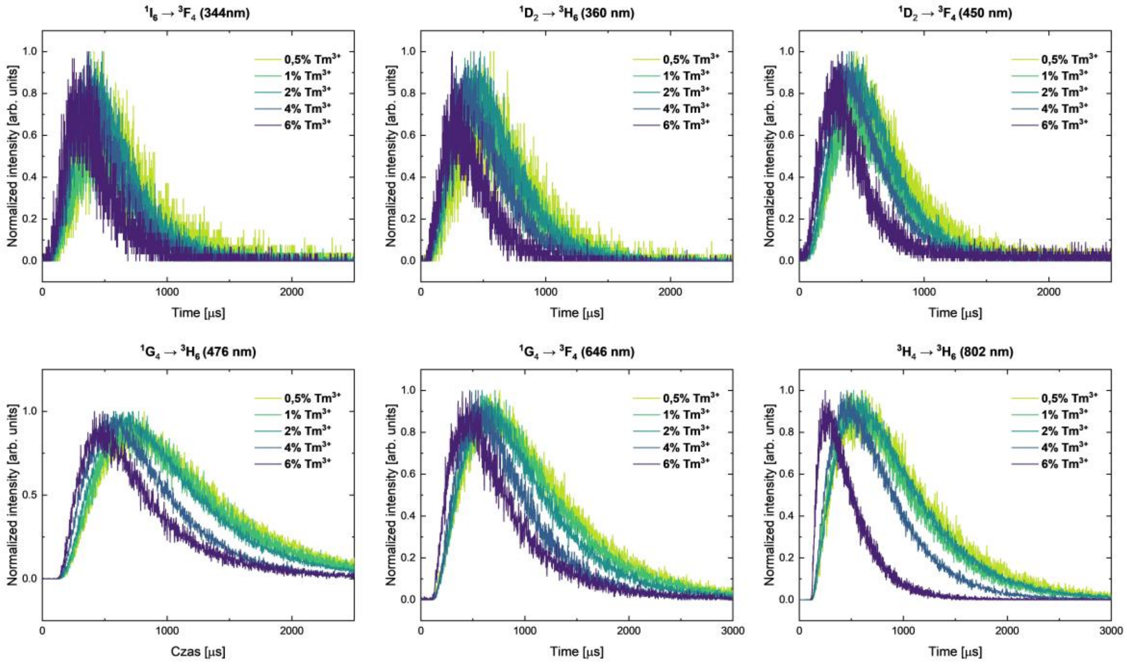

Figure S5. Decay curves of different energy levels of Tm^{3+} ions (λ_{exc} = 980 nm).

5.2. Luminescence lifetimes of nanoparticles dispersed in chloroform

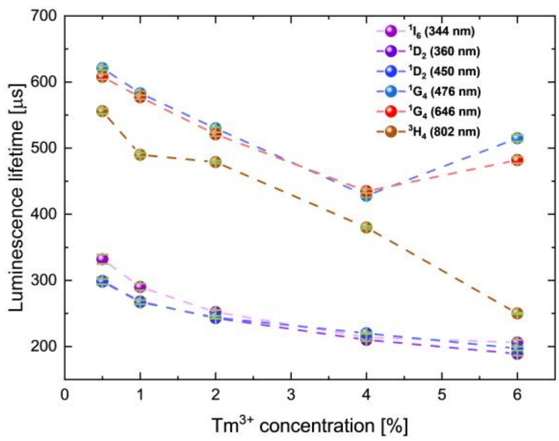

Figure S6. Luminescence lifetime dependences on Tm^{3+} concentration.

6. Förster distance

6.1 Dye structures

Table S1. Dye molecular structures

Name	Chemical structure
**ATTO 465**	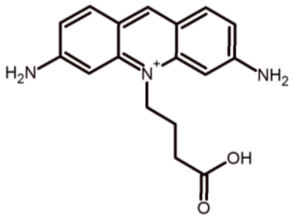
**ATTO 488**	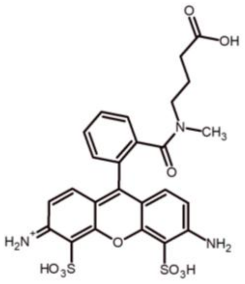
**ATTO 490LS**	Structure was not shown by producer (ATTO-TEC) at the day of this paper publication. This dye possess negative charge of -1, and it is equipped with carboxy group.
**ATTO 532**	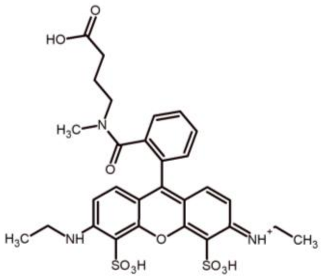

6.2 Calculation of Förster distance

In order to choose four ATTO dyes for multiplexing applications using upconverting nanoparticles we first calculated the spectral overlap (J) and Förster distance (R_0), which is a distance between donor and acceptor for which FRET efficiency is equal to 50%.[1] The higher J and R_0, the dye should be more effective acceptor in FRET, thus higher emission signal should be observed.

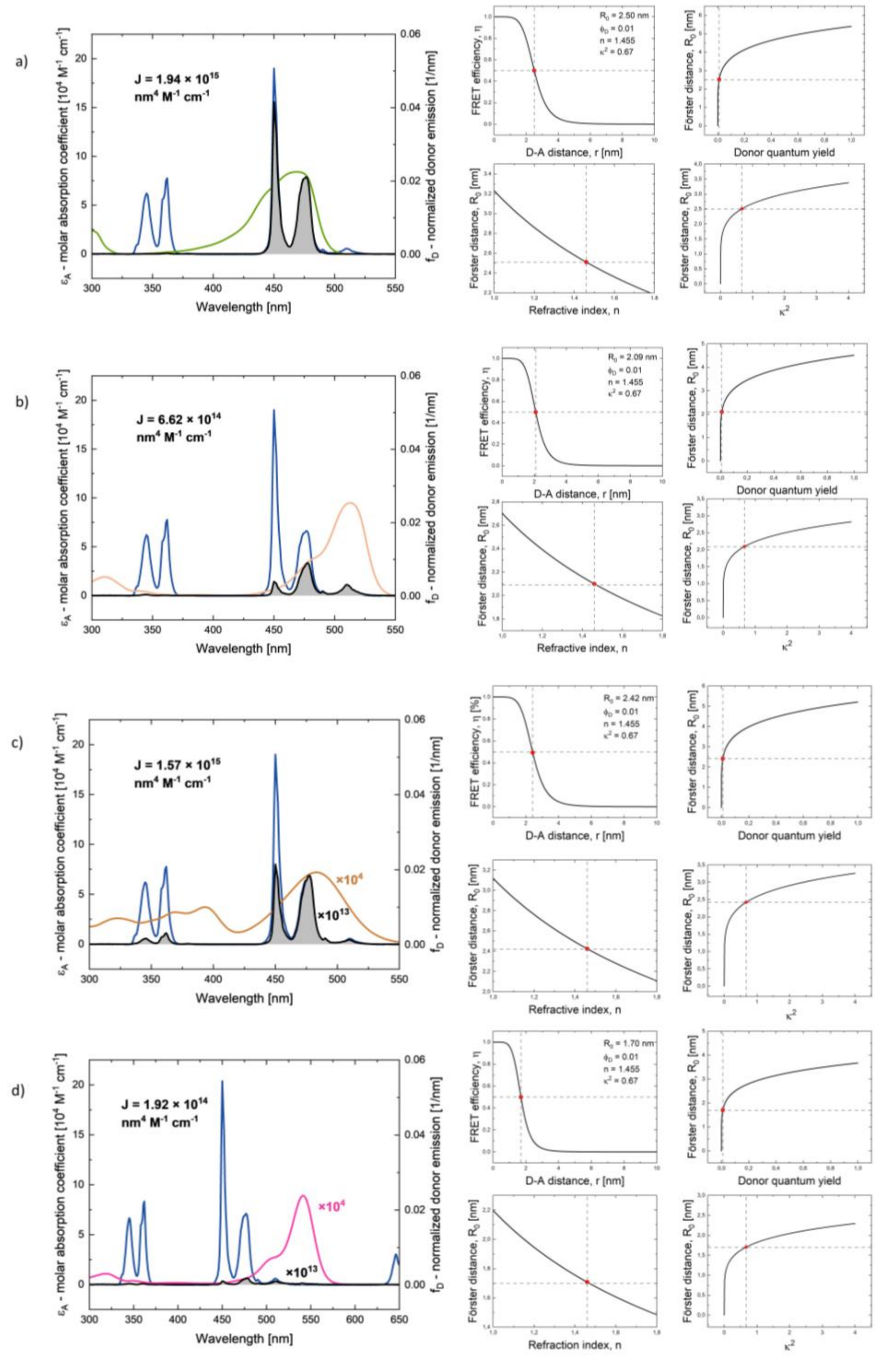

Figure S7. Spectral overlap of UCNPs emission and dye absorption, calculation of Förster distance a) ATTO465, b) ATTO 488, c) ATTO 490LS, d) ATTO 532 and its dependence on donor quantum yield, refraction index and orientation factor.

Table S2. Calculation parameters and Förster distance for each dye.

Dye	ϕ_D	n	κ^2	Förster distance [nm]
ATTO 465	0.01	1.465	0.67	2.50
ATTO 488				2.09
ATTO 490LS				2.42
ATTO 532				1.70

For calculation of FRET efficiency, Förster distance and spectral overlap we used following equations:

FRET efficiency:

$$\boldsymbol{\eta} = \frac{\mathbf{1}}{\mathbf{1}+\left(\frac{\boldsymbol{r}}{\boldsymbol{R_0}}\right)^{\mathbf{6}}} \qquad \text{Eq. S1}$$

Förster distance:

$$\boldsymbol{R_0} = \frac{\mathbf{9}\, \boldsymbol{ln}\, \mathbf{10} \cdot \boldsymbol{\varphi_D} \cdot \boldsymbol{\kappa}^{\mathbf{2}} \cdot \boldsymbol{J(\lambda)}}{\mathbf{128} \cdot \boldsymbol{\pi}^{\mathbf{5}} \cdot \boldsymbol{N_A} \cdot \boldsymbol{n}^{\mathbf{4}}} \qquad \text{Eq. S2}$$

Spectral overlap:

$$\boldsymbol{J(\lambda)} = \int \boldsymbol{f_D(\lambda)} \cdot \boldsymbol{\varepsilon_A(\lambda)} \cdot \boldsymbol{\lambda}^{\mathbf{4}}\, \boldsymbol{d\lambda} \qquad \text{Eq. S3}$$

Normalized donor emission:

$$\boldsymbol{f_D} = \frac{\boldsymbol{F_D}}{\int \boldsymbol{F_D(\lambda)}\, \boldsymbol{d\lambda}} \qquad \text{Eq. S4}$$

Where,

r – actual distance between donor and acceptor [nm]

R_0 – Förster distance [nm]

φ_D – estimated donor quantum efficiency

κ^2 – orientation factor

$J(\lambda)$ – spectral overlap [nm^4 M^{-1} cm^{-1}]

N_A – Avogadro's number [$6.02 \cdot 10^{23}$ 1/mol]

n – refractive index, calculated as a mean value of solvent (DMF: n =1.43) and nanocrystals ($NaYF_4$: n = 1.48) refractive index[2,3]

$f_D(\lambda)$ – normalized donor emission [1/nm]

$\varepsilon_A(\lambda)$ – molar absorption coefficient [M^{-1} cm^{-1}]

λ – wavelength [nm]

F_D – donor emission spectrum

6.3 Donor quantum yield discussion

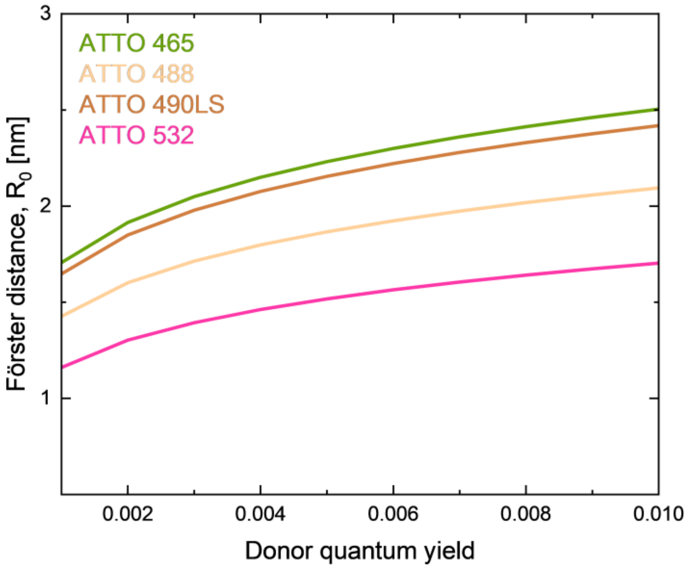

Figure S8. Förster distance dependencies on donor nanoparticles quantum yield in range from 0.1% to 1%.

Table S3. Changes in Förster distance

Dye	Minimal R_0 [QY = 0.1%]	Maximal R_0 [QY = 1%]
ATTO 465	1.7 nm	2.5 nm
ATTO 488	1.4 nm	2.1 nm
ATTO 490LS	1.6 nm	2.4 nm
ATTO 532	1.2 nm	1.7 nm

The donor quantum yield of 1% (Table S1) was estimated based on values reported for Tm^{3+}-doped $NaYF_4$ UCNPs presented by Kraft et. al.[4] However, it is not easy to directly compare UCNPs of different chemical architecture (core vs. core@shell). Furthermore, we note that methods for measuring quantum yield of UCNPs, particularly for Tm^{3+}-doped systems, are inherently approximate often due to low emission intensities, and sensitivity to particle size, dopant concentration, and surface effects. Upconversion quantum yield (UCQY) measurements are also technically demanding as lanthanides exhibit low absorption coefficients, UCQY is pump power dependent and UCQY itself is often very low, typically below 3%. Moreover, total quantum yield in whole visible spectral region differs from quantum yield of each particular emission band. The precise evaluation of quantum yield would require its values for 344 nm, 360 nm, 450 nm, 476 nm as those bands overlap with dye absorption spectrum. Although it is hard to estimate quantum yield of Tm^{3+} - doped UCNPs one can calculate Förster distance in function of donor quantum yield in a quantum yield range of 0.1% to 1%. Please note that the tendency in terms of the highest Förster distance and acceptor emission remains the same. If R_0 is larger, more Tm^{3+} ions can interact with surface-bound dye molecules resulting in efficient energy transfer, thus indicating that for ATTO 532 its signal is the weakest due to lowest R_0 and less effective energy transfer when compared with e.g. ATTO 488.

7. Dye absorption spectra

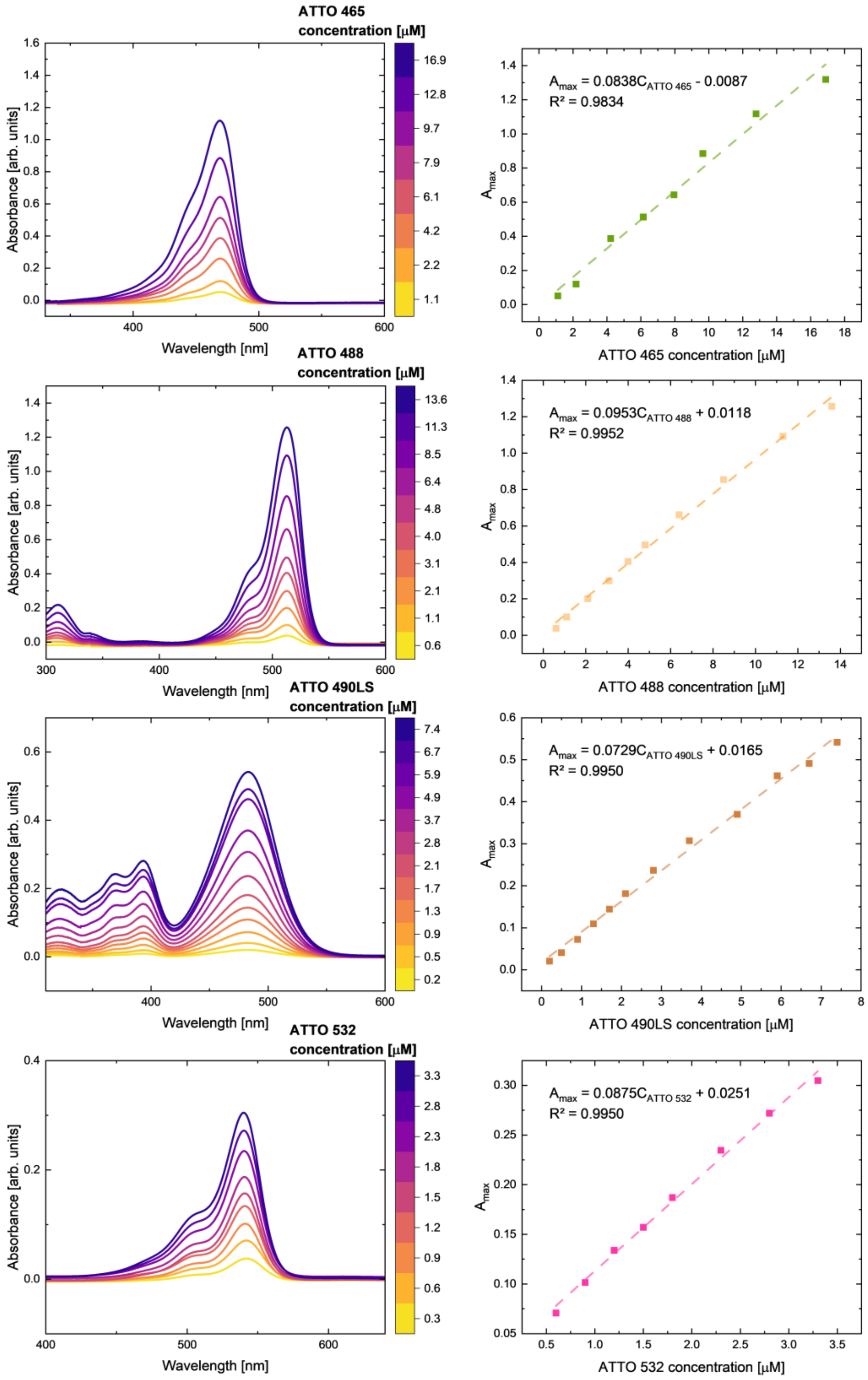

Figure S9. Absorption spectra of dyes measured in DMF using spectrophotometer Cary Varian 5E UV–vis–NIR based on which molar absorption coefficients were estimated following Lambert's-Beer's law.

8. Experimental data for optimization of Tm^{3+} concentration – emission spectra

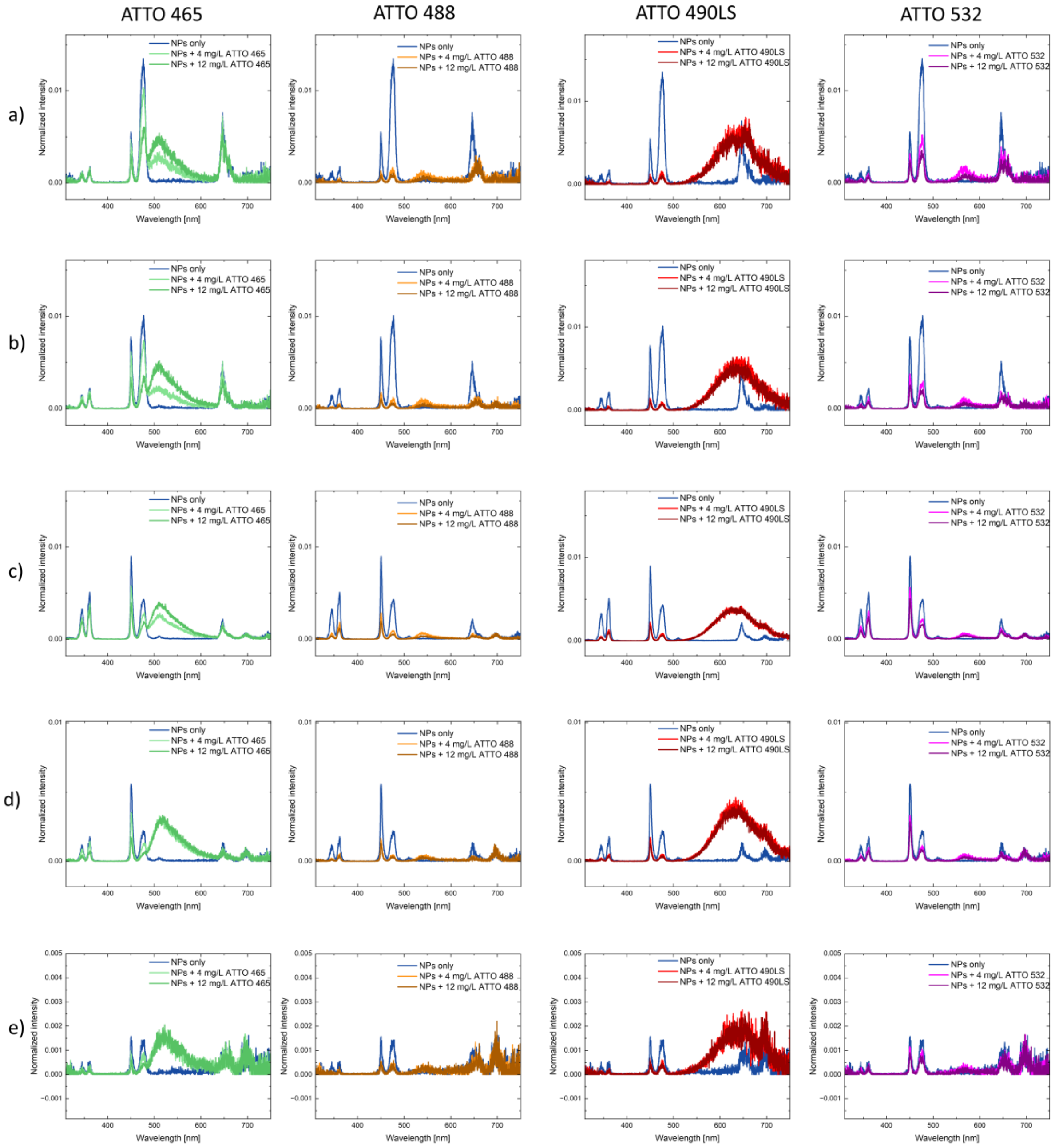

Figure S10. Normalized emission spectra of $NaYF_4$:50%Yb^{3+}@$NaYF_4$:20%Yb^{3+}, x%Tm^{3+} core@shell nanocrystals where x is equal to a) 0.5% Tm^{3+}, b) 1% Tm^{3+}, c) 2% Tm^{3+}, d) 4% Tm^{3+}, e) 6% Tm^{3+} conjugated with two different dye concentrations, measured under 980 nm laser excitation at power density of 200 W/cm^2.

9. Emission-based FRET efficiency at 4 mg/L dye concentration

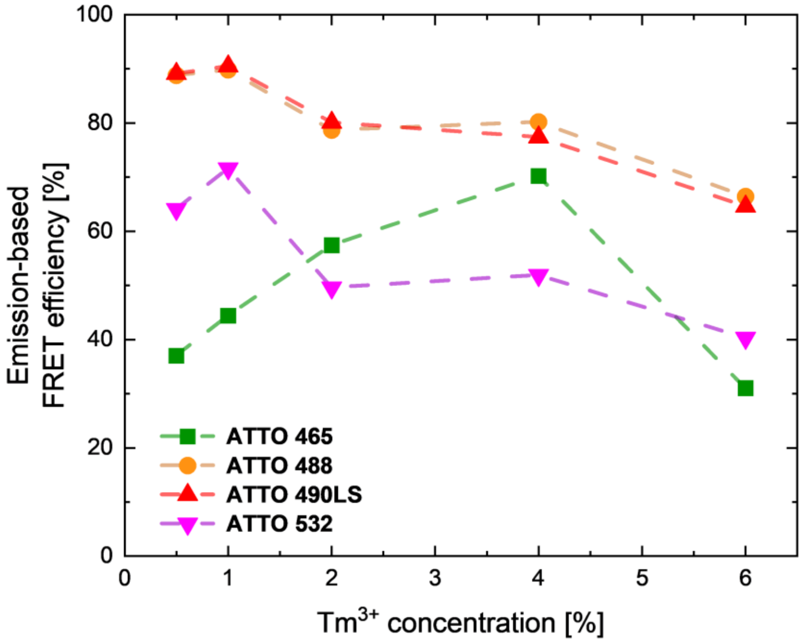

Figure S11. Energy transfer efficiency for all synthesized nanocrystals conjugated with different dye molecules at concentration of 4 mg/L based on emission intensity of donor.

10. Experimental data for optimization of Tm^{3+} concentration – luminescence lifetimes

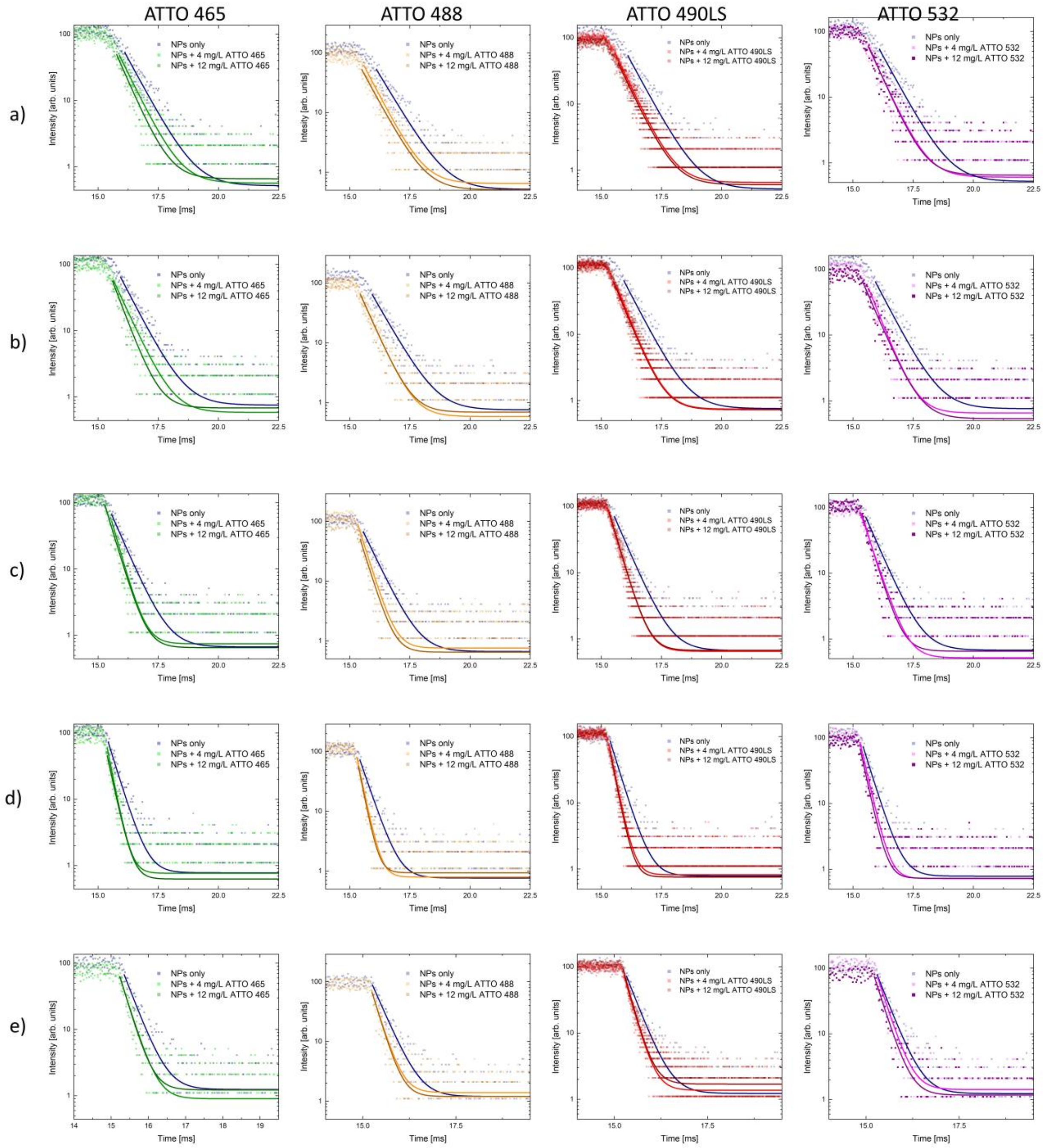

Figure S12. Kinetic profiles (λ_{exc} = 980 nm) of the $NaYF_4$:50%Yb^{3+}@$NaYF_4$:20%Yb^{3+}, x%Tm^{3+} core@shell nanocrystals where x is equal to a) 0.5% Tm^{3+}, b) 1% Tm^{3+}, c) 2% Tm^{3+}, d) 4% Tm^{3+}, e) 6% Tm^{3+} upon addition of two concentration of different dye concentration upon 980 nm laser excitation.

11. Lifetime-based FRET efficiency at 4 mg/L dye concentration

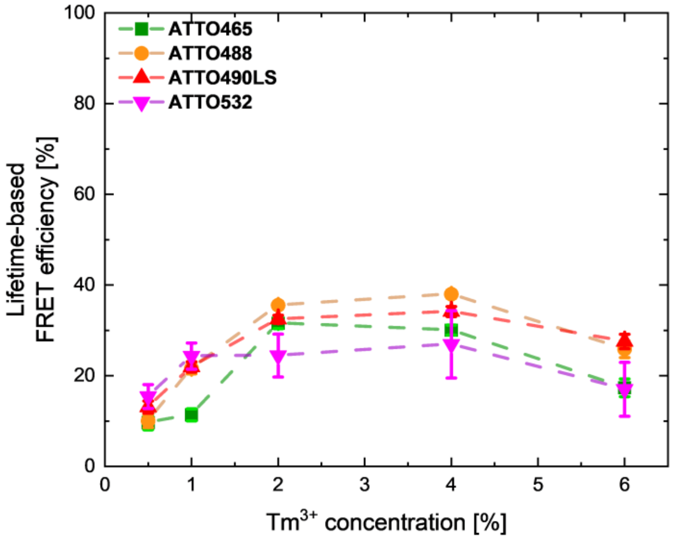

Figure S13. FRET efficiency based on luminescence lifetimes of Tm^{3+} $^1G_4$ energy level in the presence of acceptor molecules at concentration of 4 mg/L.

12. Analysis of donor emission in the presence of acceptor

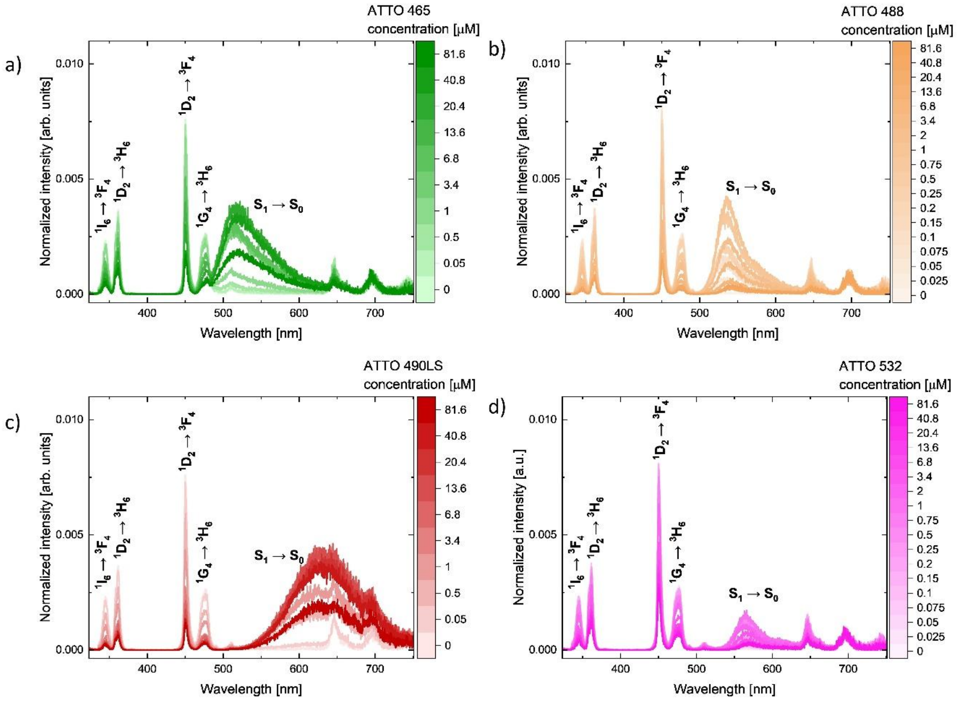

Figure S14. Normalized emission spectra (λ_{exc} = 980 nm, I_p = 200 W/cm^2) of core@shell $NaYF_4$:50%Yb^{3+}@$NaYF_4$:20%Yb^{3+}, 4% Tm^{3+} nanoparticles conjugated with a) ATTO 465, b) ATTO 488, c) ATTO 490LS and d) ATTO 532 dyes.

13. Investigation of dye aggregates formation

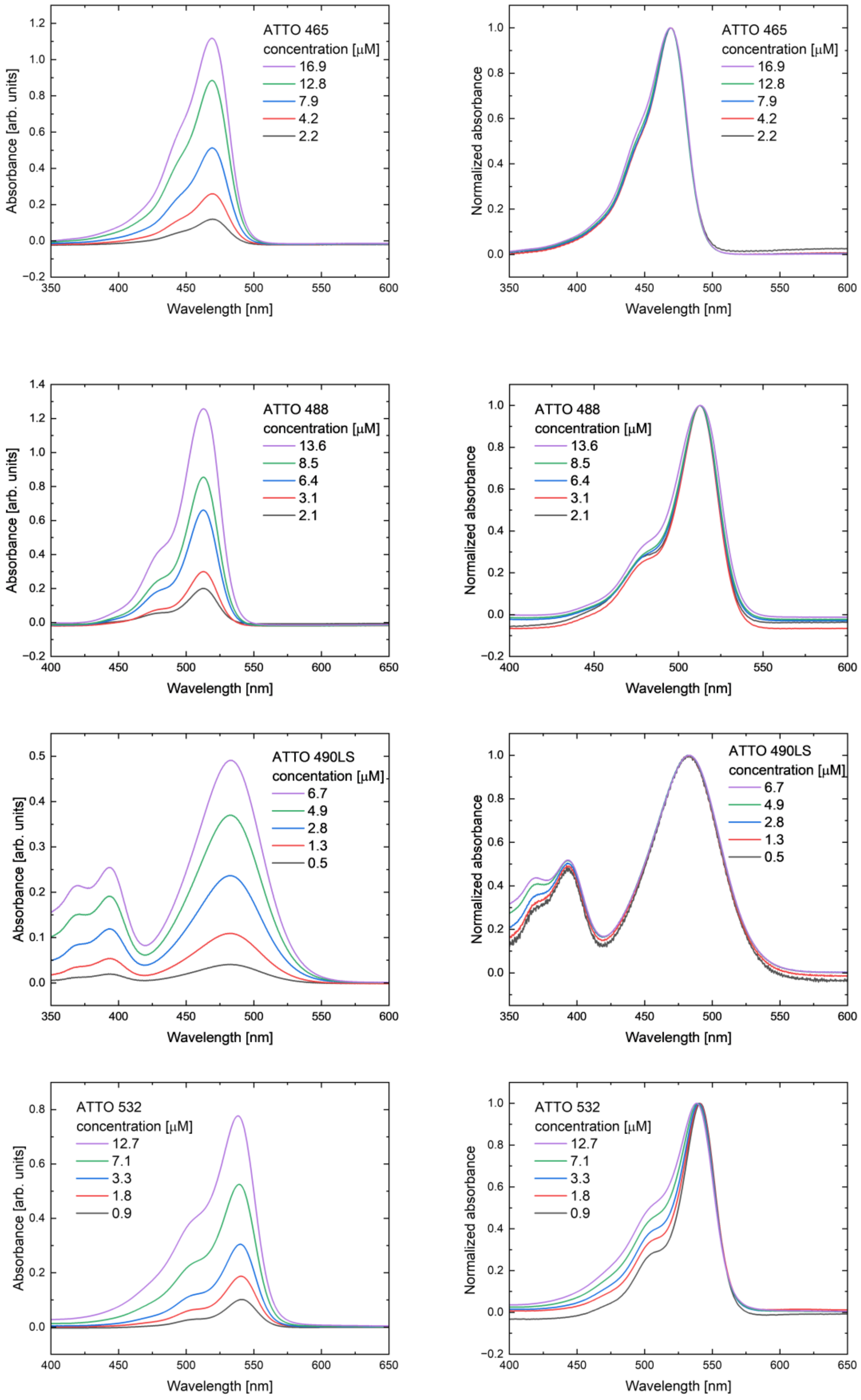

Figure S15. Absorption spectra of analyzed ATTO dyes in DMF solution – relative intensities (right) and intensity normalized to absorption band at longer wavelength (left).

14. Analysis of luminescence lifetimes of $^1G_4$ energy level in the presence of acceptor

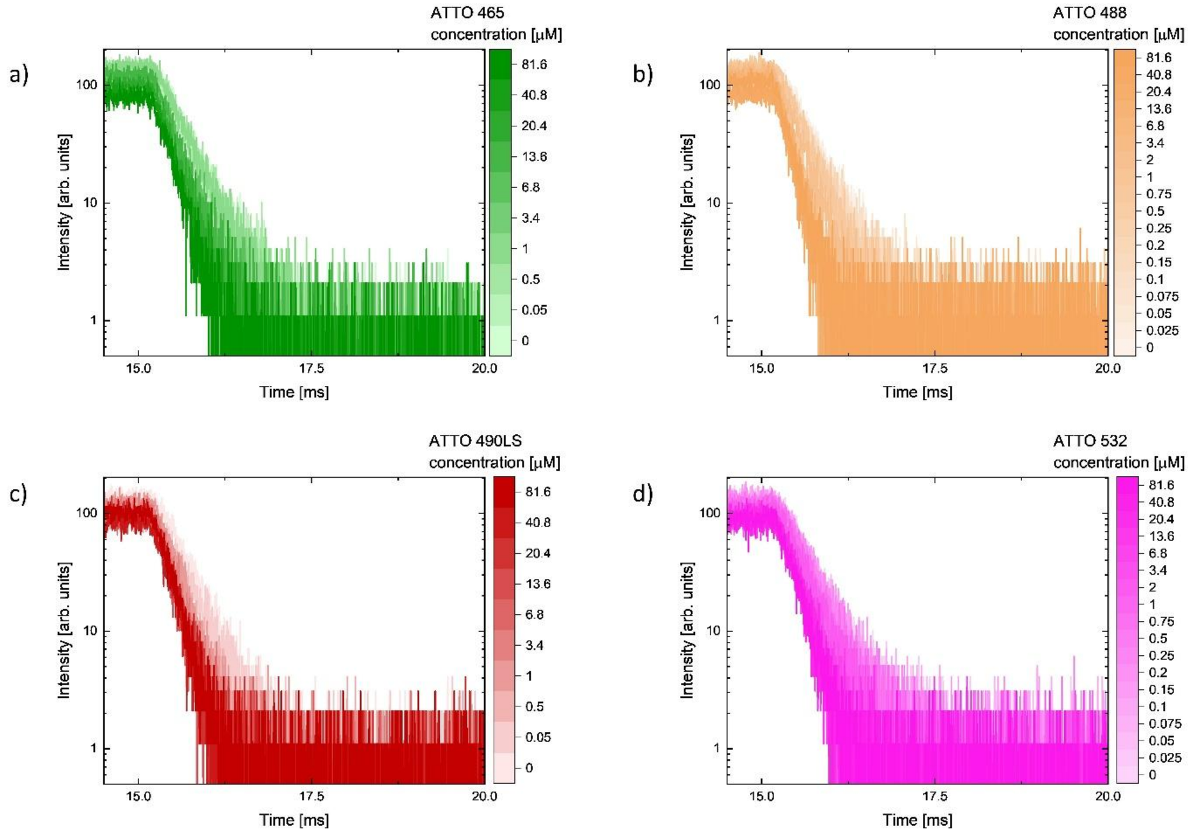

Figure S16. Luminescence kinetics (λ_{exc} = 980 nm) of core@shell $NaYF_4$:50%Yb^{3+}@$NaYF_4$:20%Yb^{3+}, 4% Tm^{3+} ($^1G_4$ energy level) nanoparticles conjugated with a) ATTO 465, b) ATTO 488, c) ATTO 490LS and d) ATTO 532 dyes.

Error of obtained FRET efficiency based on luminescence lifetimes derived from aforementioned decay curves of $^1G_4$ energy level in presence of acceptor was calculated as follows:

$$\sigma_\eta = \sqrt{\left(\frac{\partial\eta}{\partial\tau_{DA}} \cdot \sigma_{\tau_{DA}}\right)^2 + \left(\frac{\partial\eta}{\partial\tau_D} \cdot \sigma_{\tau_D}\right)^2}$$

Where η is FRET efficiency, τ_{DA} is luminescence lifetime of $^1G_4$ energy level of donor in the presence of acceptor, $\sigma_{\tau_{DA}}$ is a deviation obtained from fitted decay curve, τ_D is luminescence lifetime of $^1G_4$ energy level of donor in the absence of acceptor, σ_{τ_D} is a deviation obtained from fitted decay curve

Fitting decay curves three times let us eliminate operator error which depends on fitting range.

15. Estimation of LOD

Limit of detection (LOD) is the minimal amount of analyte that can be detected using certain method. It can be estimated using following equation:

$$LOD = \ y_0 + 3\sigma \qquad \text{Eq. 5}$$

Where y_0 is defined here as a signal (LIR_1) from UCNPs that are not conjugated with dye molecules, and σ is a standard deviation of that value.

15.1 LIR_1 dose – response curves

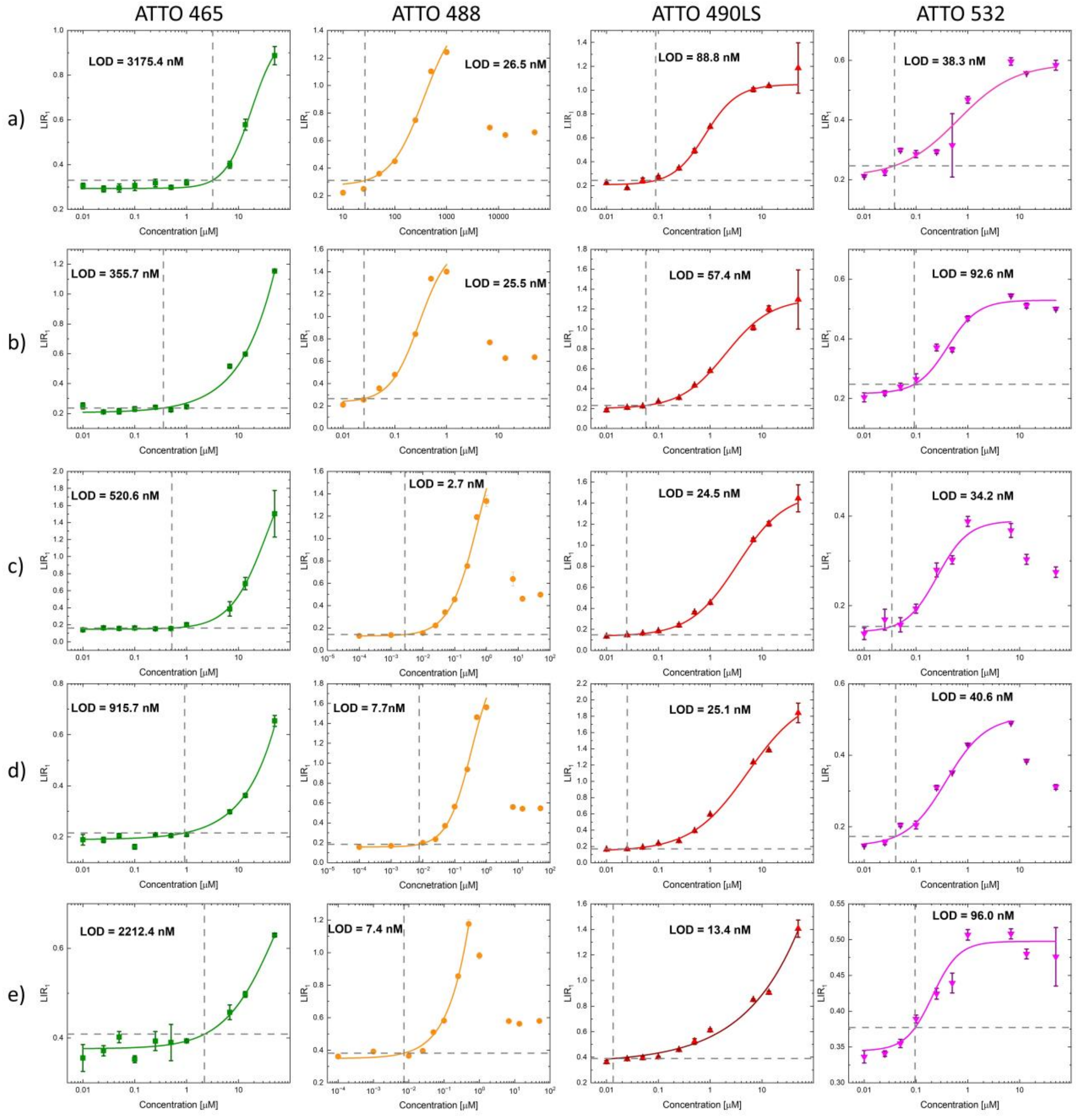

Figure S17. LIR_1 curves for estimating LODs of $NaYF_4$:50%Yb^{3+}@$NaYF_4$:20%Yb^{3+}, x%Tm^{3+} core@shell nanocrystals where x is equal to a) 0.5% Tm^{3+}, b) 1% Tm^{3+}, c) 2% Tm^{3+}, d) 4% Tm^{3+}, e) 6% Tm^{3+} conjugated with four different ATTO dyes.

15.2 Summary of LODs

Table S3. LODs estimated for synthesized nanocrystals conjugated with ATTO dyes.

$NaYF_4$:50%Yb^{3+} @ $NaYF_4$:20%Yb^{3+}, x%Tm^{3+}	LOD [nM]			
	ATTO465	ATTO488	ATTO490LS	ATTO532
x = 0.5%	3175.4	26.5	88.8	38.3
x = 1%	355.7	25.5	57.4	92.6
x = 2%	520.6	2.7	24.5	34.2
x = 4%	915.7	7.7	25.1	40.6
x = 6%	2212.4	7.4	13.4	96.0

16. Calculation of dye molecules per nanocrystal

For dye conjugation we used 5 µl of 80 mg/mL stock solution of nanoparticles in DMF. This means that the mass of UCNPs is equal to 4 x 10^{-4} g. Molar mass of $NaYF_4$:50%Yb^{3+}@$NaYF_4$:20%Yb^{3+}, 4%Tm^{3+} can be calculated in a following way (as presented by Zhou, Jian, et al.[5]):

Avogadro's number: N_A = 6.022 x 10^{23} mol^{-1}

Core radius R_c = 16.3 nm

Core@shell radius R_{cs} = 18.5 nm

Therefore volume ratio of shell to core can be calculated as:

$$\frac{V_{shell}}{V_{core}} = \left(\frac{R_{cs}}{R_c}\right)^3 - 1 = 0.46$$

A β-$NaLnF_4$ unit cell is composed of 1.5 Na atoms, 1.5 Ln atoms and 6 F atoms, which can be written as $Na_{1.5}Ln_{1.5}F_6$. Using calculated volume ratio of shell to core, single UCNP composition can be written as unit cell form:

$$Na_{1.5}Y_{\left(\frac{0.5+0.76\cdot 0.46}{1+0.46}\right)\cdot 1.5}Yb_{\left(\frac{0.5+0.2\cdot 0.46}{1+0.46}\right)\cdot 1.5}Tm_{\left(\frac{0.04\cdot 0.46}{1+0.46}\right)\cdot 1.5}F_6$$

Thus we obtain:

$$Na_{1.5}Y_{0.8729}Yb_{0.6082}Tm_{0.0189}F_6$$

And relative molecular mass of singe UCNP can be calculated as:

$$M_{cell} = \frac{1.5 \cdot M_{Na} + 0.8729 \cdot M_{Y} + 0.6082 \cdot M_{Yb} + 0.0189 \cdot M_{Tm} + 6 \cdot M_{F}}{N_A} = 5.56 \cdot 10^{-22} g$$

Volume of $NaLnF_4$ unit cell, according to ICSD-51917 database file is equal to 1.09 x 10^{-22} cm^3, so the density of core@shell nanoparticles can be calculated as:

$$\rho = \frac{M_{cell}}{V_{cell}} = 5.1 \frac{g}{cm^3}$$

So the mass of single UCNPs is equal to:

$$m_{single\ UCNP} = \rho \cdot V_{cs} = \rho \cdot \frac{4}{3} \pi R_{cs}^3 = 1.11 \cdot 10^{-16} g$$

Thus the number of nanoparticles is equal to:

$$N_{NCs} = \frac{4 \cdot 10^{-4}}{m_{single\ UCNP}} = 3.6 \cdot 10^{12}$$

Table S5. Number of dye molecules at given concentration

Dye concentration [µM]	Number of dye molecules
0	0
0.025	7.53E+12
0.05	1.51E+13
0.075	2.26E+13
0.1	3.01E+13
0.15	4.52E+13
0.2	6.02E+13
0.25	7.53E+13
0.5	1.51E+14
0.75	2.26E+14
1	3.01E+14
2	6.02E+14
3.4	1.02E+15
6.8	2.05E+15
13.6	4.09E+15
20.4	6.14E+15
40.8	1.23E+16
81.6	2.46E+16

Table S6. Number of dye molecules per one nanocrystal at given dye concentration

Dye concentration [µM]	Number of dye molecules per 1 nanocrystal
0	0
0.025	2
0.05	4
0.075	6
0.1	8
0.15	13
0.2	17
0.25	21
0.5	42
0.75	63
1	84
2	167
3.4	284
6.8	569
13.6	1137
20.4	1706
40.8	3411
81.6	6823

17. Photostability of UCNPs conjugated with dye molecules

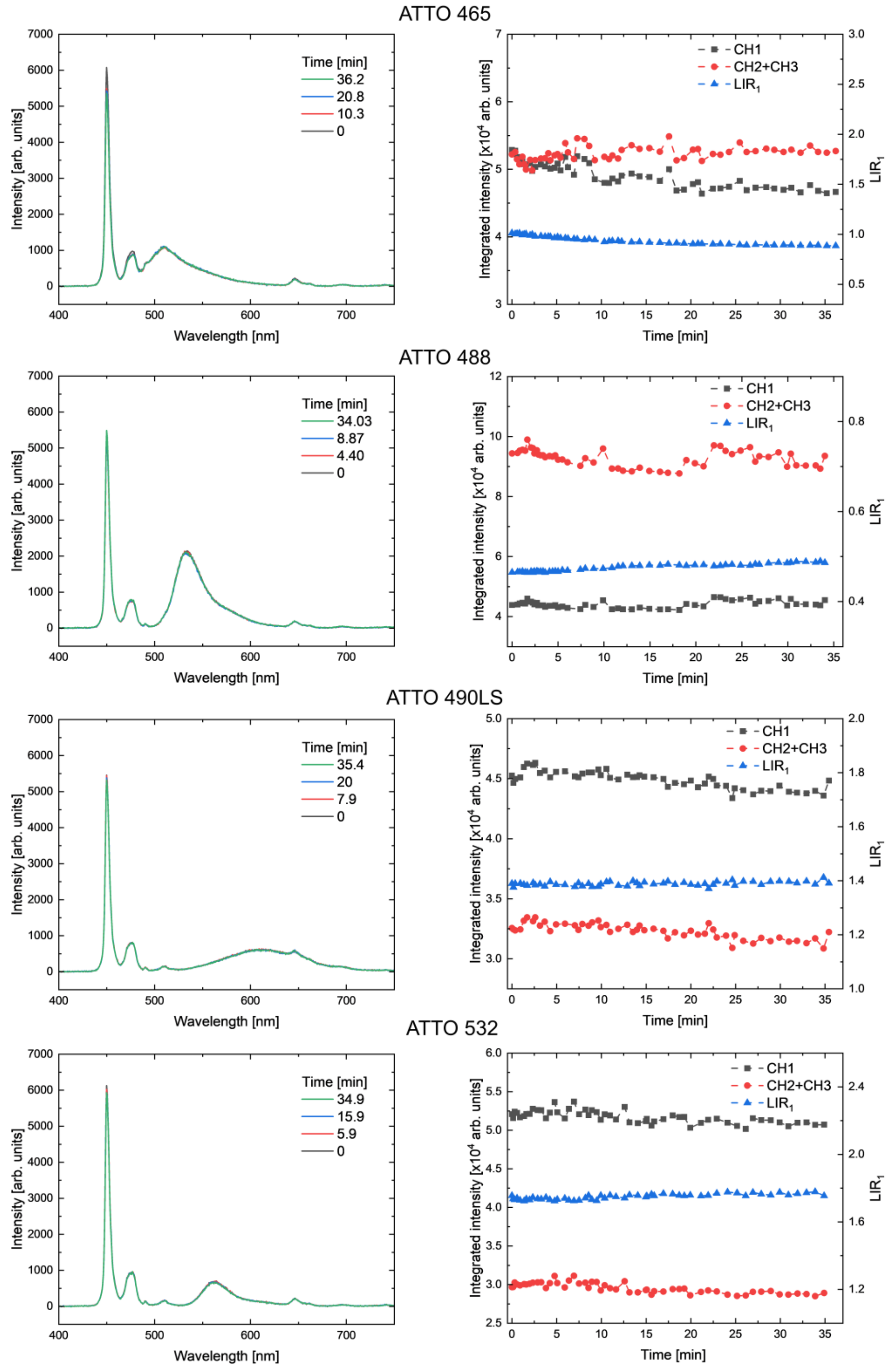

Figure S18. Emission spectra at given time of UCNPs conjugated with dye molecules (I_p = 200 W/cm^2; left) and changes in CH1, CH2+CH3 spectral regions, and in LIR$_1$.

Photostability of investigated systems is shown based on their emission spectra under continuous wave 980 nm photoexcitation (I_p = 200 W/cm^2; same experimental conditions as during previous FRET measurements) for more than 30 minutes. Left figures show emission spectra after irradiation for given time and right – intensity of Tm^{3+} emission (integrated in the range of channel CH1 = 435 – 485 nm),

intensity of dye emission (integrated in the range of channel CH2+CH3 = 500 – 614 nm) and ratio between CH2+CH3 and CH1 (which is LIR_1 ratio in this paper).

Based on obtained spectra and relation between emission intensity of Tm^{3+} and dye channels we can state that in the course of more than 30 minutes no visible dye photobleaching is detected. Some fluctuations can be prescribed to fluctuations in laser diode or spectrophotometer work and decrease in Tm^{3+} emission intensity also resulted in decrease of dye emission, while the ratio between them remains the same. More pronounced deviations can be observed when ATTO 465 molecules are conjugated to UCNPs. Due to the overlap of this dye emission and Tm^{3+} emission in the CH1 region the decrease over time resulted in the highest 11% change of emission intensity when compared to t = 0 min value. However, after 5 minutes of irradiation this change is equal to only 3% thus, we can conclude that when previous measurement lasted around one minute, impact of photodegradation or photobleaching can be ruled out. Furthermore, during the measurements, the UCNPs-dye conjugates were continuously mixing by convection in the colloidal solution. Since only a small portion of the sample volume was directly irradiated by thin laser beam and given that the dyes are not expected to photobleach rapidly, no significant changes in their photophysical properties should occur.

18. References

(1) Medintz, I. L. .; Hildebrandt, Niko. FRET - Förster Resonance Energy Transfer : From Theory to Applications; Wiley-VCH Verlag GmbH, 2014.

(2) Sokolov, V. I.; Zvyagin, A. V.; Igumnov, S. M.; Molchanova, S. I.; Nazarov, M. M.; Nechaev, A. V.; Savelyev, A. G.; Tyutyunov, A. A.; Khaydukov, E. V.; Panchenko, V. Y. Determination of the Refractive Index of β-NaYF4/Yb3+/Er3+/Tm3+ Nanocrystals Using Spectroscopic Refractometry. *Opt. Spectrosc.* **2015**, 118 (4), 609–613.

(3) https://www.sigmaaldrich.com/PL/pl/product/mm/102375?srsltid=AfmBOookecGwnTbhAMpKznydN723CZJjA1XqHUv3eTysSbFRMhINB-ID.

(4) Kraft, M.; Würth, C.; Palo, E.; Soukka, T.; Resch-Genger, U. Colour-Optimized Quantum Yields of Yb, Tm Co-Doped Upconversion Nanocrystals. *Methods Appl. Fluoresc.* **2019**, 7 (2), 024001.

(5) Zhou, J.; Li, C.; Li, D.; Liu, X.; Mu, Z.; Gao, W.; Qiu, J.; Deng, R. Single-Molecule Photoreaction Quantitation through Intraparticle-Surface Energy Transfer (i-SET) Spectroscopy. *Nat. Commun.* **2020**, 11 (1), 4297.
